# Genetic Architecture of Flowering Time and Sex Determination in Hemp (*Cannabis sativa* L.): A Genome-Wide Association Study

**DOI:** 10.3389/fpls.2020.569958

**Published:** 2020-11-04

**Authors:** Jordi Petit, Elma M. J. Salentijn, Maria-João Paulo, Christel Denneboom, Luisa M. Trindade

**Affiliations:** ^1^Wageningen UR Plant Breeding, Wageningen University and Research, Wageningen, Netherlands; ^2^Biometris, Wageningen University and Research, Wageningen, Netherlands

**Keywords:** GWAS, *Cannabis sativa*, hemp, plant breeding, sex determination, flowering time, QTL

## Abstract

Flowering time and sex determination in hemp (*Cannabis sativa* L.) strongly influence fiber quality and seed production of this crop. The control of these traits is paramount for the breeding of new cultivars. Yet, little is known about the genetics underlying such complex traits and a better understanding requires in depth knowledge of the molecular mechanisms responsible for these traits. In this report, the genetic architecture of flowering time and sex determination in hemp was studied using a Genome-Wide Association Studies (GWAS) approach. Association studies were performed on a panel of 123 hemp accessions, tested in three contrasting environments, using a set of 600 K SNP markers. Altogether, eight QTLs were identified across environments; six for flowering time traits and two for sex determination. These QTLs covered genomic regions with 33 transcripts predicted to be involved in flowering and sex determination as well as a microRNA, *miR156.* Genes related to perception and transduction of light and transcription factors well-known to regulate flowering were identified in QTLs for flowering time traits. Transcription factors and genes involved in regulating the balance of phytohormones, specially auxins and gibberellic acid, were identified in QTLs for sex determination. Sex determination QTLs were associated with the development of male flowers in female plants and thus with the stability of sex determination in monecious plants. The present study elucidates relevant knowledge on the genetic mechanisms of flowering and sex determination traits in hemp, and provides new tools for hemp breeding.

## Introduction

Hemp (*Cannabis sativa* L.) is naturally a diecious species with distinct male- and female plants. The sexual phenotype of *Cannabis* frequently shows some flexibility leading to differentiation of hermaphrodite plants, also known as monecious phenotypes (Moliterni et al., 2004). The species is a short-day plant characterized by sexual dimorphism. True males can be recognized by their typical morphology characterized by slender stature, few leaves and hanging inflorescences carrying male flowers. Female plants produce female pistillate flowers in dense panicles heads interspaced with leafy bracts. Monecious plants morphology resembles that of female plants (Faux et al., 2016). Male plants mostly flower earlier than female plants (Bócsa and Karus, 1998; Struik et al., 2000) and die shortly after flowering, while female plants remain alive until seed maturation.

The post-emergent growing period of hemp is divided into a vegetative phase and a flower development phase. The vegetative phase is divided into a temperature-dependent basic vegetative phase and a daylength-dependent photoperiod induced phase (Lisson et al., 2000). Hemp is strongly sensitive to changes in the photoperiod regime and temperature input (Amaducci et al., 2008a, 2012; Salentijn et al., 2015, 2019; Petit et al., 2020b). Flowering time of hemp rapidly responds to these environmental variations to synchronize the flowering with the environment, especially with the daylength of the growing season (Amaducci et al., 2012; Salentijn et al., 2019). Daylength has a key influence on the timing of flowering in hemp (Lisson et al., 2000). Hemp flowering is generally inhibited during long-day photoperiod regimes and is induced promptly when the photoperiod changes to short-day regimes with a threshold of ∼10–12 h of uninterrupted darkness (critical photoperiod of ∼12–14 h of daylight) (Salentijn et al., 2019). However, Schaffner (1926) observed that hemp grown under 24 h daylength also flowered, but with a strong reduction of dry matter in the floral parts. Similarly, Van der Werf et al. (1994) reported hemp plants of cultivar Fedrina 74 and Kompolti Hybrid TC that flowered under continuous illumination (24 h). The initiation of flowering independently of the photoperiod is referred to as “autoflowering” or day neutral. Some cannabis cultivars, especially for cannabinoid production, such as *C. sativa* subsp. *sativa* var. spontanea, have been described as day neutral plants [reviewed in McPartland (2018)]. Consequently, all hemp cultivars are considered as quantitative short-day plants (Lisson et al., 2000), since they have different sensitivity to photoperiod. A recent study described large quantitative variation of flowering time in a panel of 123 accessions (Petit et al., 2020b). To simplify, breeders classify hemp cultivars in early, middle and late flowering cultivars (Amaducci et al., 2015; Salentijn et al., 2015). Despite the large variation in flowering time and the extreme phenotypes (day neutral), if the critical short-day regime is not reached within the growing season or if cultivars are very late flowering, hemp plants generally remain vegetative until harvesting time (Lisson et al., 2000; Amaducci et al., 2008a, b; Petit et al., 2020b). In hemp, temperature regime is of special importance during the basic vegetative phase (juvenile stage), since it is dependent on the temperature (Lisson et al., 2000). The completion of the basic vegetative phase requires a certain temperature input, before entering to the daylength-dependent photoperiod induced phase. Hemp growth requires a base air temperature of around 1°C, with an optimal temperature for growth of 29°C and a ceiling temperature 41°C. A range of 306–636°Cd (accumulated thermal time over a period with a base temperature of 1°C) are required for completion of the basic vegetative stage and beginning the daylength-dependent induced phase [references in Amaducci et al. (2008a, 2012) and Salentijn et al. (2019)].

Hemp is a diploid species with 9 pairs of homomorphic autosomal chromosomes and a pair of heteromorphic sex chromosomes: X and Y (2*n* = 20) (Moliterni et al., 2004). The haploid genome size is 818 Mbp for female plants and 843 Mbp for male plants (van Bakel et al., 2011). Sex determination system in diecious hemp has been well studied. Male plants carry the heterogametic sex (XY) and female plants the homogametic one (XX). However, despite the presence of specific sex chromosomes, the phenotypic expression of sex in hemp shows some flexibility. Some diecious hemp plants produce flowers of the opposite sex than the one determined by their chromosomal composition (Moliterni et al., 2004). Monecious hemp plants carry the homogametic sex (XX) and the ratio of female to male flowers in a single monecious plant is highly variable (Faux et al., 2014). This variation ranges from monecious plants that have predominantly male flowers to predominantly female flowers (Faux et al., 2013, 2014, 2016). Diecious hemp species abundantly exist in nature, while monecious plants have been developed from some mutants that were selected during the domestication of the crop. Monecious accessions tend to show a wide range in sex ratios within the crop, including unisexual plants, and may gradually return to natural dioecy after a few generations (Bócsa and Karus, 1998; Amaducci et al., 2008a; Faux et al., 2013, 2014; Faux and Bertin, 2014). Constant strict selection of monecious plants is therefore needed to maintain monoecy during the seed multiplication (Moliterni et al., 2004). The instability of the sexual phenotype across generations, and the quantitative nature of expression of the sex suggests that sex expression is a rather polygenic trait (Faux et al., 2013, 2014; Faux and Bertin, 2014).

Genetic variation of sex expression among cultivars in hemp has been observed (Moliterni et al., 2004; Faux et al., 2014, 2016). Moliterni et al. (2004) studied the differences in genetic expression between males and females of the diecious hemp cultivar ‘Fibranova.’ They identified polymorphic fragments of cDNA-AFLP markers that allowed to develop a male-specific SCAR marker for the early detection of male plants. Cloning and sequencing revealed that the mRNA of the AFLP markers belonged to genes involved in the activation of auxin-induced genes, probably involved in the female sex differentiation (Galoch, 1978). Faux et al. (2016) performed the first association mapping studies in three biparental hemp populations, two of diecious- and one of monecious hemp, using 71 AFLP markers. The study resulted in the identification of 5 QTLs associated with sex expression putatively located on sex chromosomes (the X chromosomes of monecious hemp and the X and Y chromosomes of diecious hemp). Four markers were found common between monecious and diecious populations, suggesting a partly common genetic basis. These results establish the prerequisite for further research on the genetic determination of sex in hemp using quantitative approaches. Furthermore, monecious accessions were partly linked to earliness, while diecious accessions to later flowering. This link suggested a partly common genetic variation and thus a partly common genetic basis underpinning the determination of sex-type (monecious/diecious) and flowering time (Faux et al., 2013; Petit et al., 2020b). Another link between sex-expression and flowering time is also found in the fact that male flowers are earlier than female flowers.

Many studies suggested that in hemp, epigenetic mechanisms could be associated with the control of sex determination, besides genetic factors [reviewed in Truta et al. (2007)]. The phenotypic outcome of sex expression in hemp might be controlled at the transcriptional- or post-transcriptional level, so without alterations in the DNA or affecting chromatin structures (Soldatova and Khryanin, 2010). The studies confirmed the role of endogenous phytohormones in the regulation of genetically determined sex type, as well as the possibility to modify it by exogenous external factors, which include ions, phytohormonal treatments, environmental factors such as photoperiod, etc [reviewed in Truta et al. (2007)]. The external factors exert a strong modifying effect, especially on the sex expression of monecious plants, in which both staminate and pistillate flowers are induced, in different proportions. The accumulation of Cu^++^ and Zn^++^ ions with zeatin induce feminization, whereas the accumulation of Pb^++^ ions favors a masculinization effect (Chailakhyan and Khryanin, 1978; Freeman et al., 1980; Galoch, 2015). The phytohormones gibberellins, auxins, ethylene and cytokinins play a role on the expression of sex in many monecious and diecious systems (Truta et al., 2007). Generally, auxins and ethylene have feminizing effects (Heslop-Harrison, 1963; Galoch, 1978), whereas cytokinins and gibberellins have masculinizing effects, though contrasting effects have been observed for some species and experimental conditions [reviewed in Ainsworth (2000)]. For example, Chailakhyan and Khryanin (1978) and Galoch (1978), cited by Dellaporta and Calderon-Urrea (1993) also reported feminizing effects of 6-benzylaminopurine (cytokinin) in species of *Vitis*, *Spinacia*, and *Cannabis*. In species such as *Mercurialis annua* and *Arabidopsis thaliana*, auxins induce expression of genes which exert a direct control on the biogenesis of sexual organs. Examples of such auxin regulated genes are *auxin responsive factor* genes (*arf* genes) in tomato, arabidopsis and papaya, which are transcription factors that control female flower development [reviewed in Li et al. (2016)]. It is possible that the gibberellins act as repressors of female flower development. The ratio of the different phytohormones is very important in the phenotypic sex expression in hemp (Truta et al., 2007). Gibberellins are for instance known to promote masculinization in spinach [reviewed in Salentijn et al. (2019)]. West and Golenberg (2018) reported that external treatment of gibberellic acid (GA) in spinach affected the expression of the *gibberellic acid insensitive* gene (*gai* gene), which is a transcription factor of the DELLA family. The *gai* gene is highly expressed in female inflorescences. GAI transcription factor is a repressor of the expression of *B-class* homeotic genes, which are masculinizing factors. *B-class* genes stimulate male organ formation and simultaneously supress the development of female organs in the flowers. This study in spinach concluded that high levels of exogenous GA inhibit the expression of *gai* gene, which consequently release inhibition on the masculinizing factors. Furthermore, the effect of photoperiod on sex determination of hemp could be associated with the external regulation of GA biosynthesis by differences in light quality and daylength (Hedden and Phillips, 2000). Schaffner (1921) reported that female hemp plants grown under low light winter conditions produced male flowers, while those plants grown under spring normal conditions did not produce male flowers. Another environmental factor is the soil quality. For example, a nitrogen-rich nutrition induces masculinization [reviewed in Truta et al. (2007)].

Hemp is a multi-purpose crop valuable for the production of fibers, cannabinoids, seeds and oils (Salentijn et al., 2015). It is particularly known to produce bast-fibers of large quality (Petit et al., 2020a, b). The transition from the vegetative to the flower development phases (flowering time) is of great relevance for hemp breeding, since maximum fiber quality occurs shortly after flowering (Meijer et al., 1995; Amaducci et al., 2008b, 2015). After this transition, the fiber quality decreases as the nutrient flow and the carbon partitioning is shifted from the stems, leaves and roots toward the development of flowers and seeds. Moreover, the flower development phase also coincides with the time-point of secondary bast fiber formation, which is characterized by intense lignification (Liu et al., 2015) and may cause a decrease in fiber quality (Mediavilla et al., 2001). Consequently, harvesting time is an important crop management factor influencing fiber quantity and quality in hemp (Westerhuis et al., 2009). The sex of the plants is another important factor influencing these traits. Large differences in the content and quality of fibers between diecious and monecious plants have been reported. A recent study showed that, monecious accessions produced on average more bast fibers, and higher levels of cellulose and mannan, while diecious accessions showed a stronger vigor in combination with later flowering and higher contents of xylans and lignin (Petit et al., 2020b). Additionally, as lignification intensifies around the onset of flowering and continues until seed maturation, female plants are more lignified than males (Liu et al., 2015). Male plants are also known to produce finer fibers than female but are more susceptible to pests (Amaducci et al., 2015). In addition, the proportion of males and females has an effect on the seed yield, as a large proportion of males is associated with seed yield reduction (Faux and Bertin, 2014). Furthermore, diecious hemp accessions are characterized by heterogeneity in fiber and seed production. In contrast, monecious accessions are more stable in both fiber and seed production because the plants develop more uniformly (Mandolino and Carboni, 2004; Salentijn et al., 2015). The differences in properties between diecious and monecious plants have traditionally led to a specialization of their applications, as sex determination of hemp strongly influences fiber quality and seed yield [reviewed in Salentijn et al., 2019]. As a consequence, diecious are mostly used for fiber production, while monecious are produced for dual purpose: fiber and seed production (Amaducci and Gusovius, 2010; Amaducci et al., 2015).

Hemp breeding goals targeted for control of flowering time and sex determination offer the potential for sustainable gains on hemp yield and quality of fibers. This will help to breed for cultivars better adapted to specific photoperiod regimes and with desired seed and/or fiber yields and qualities. For instance, early flowering under non-inductive (long-daylength) conditions and reduced sensitivity to photoperiod is interesting for hemp adaptations and essential for reproductive success and good fiber and seed yields in Northern European latitudes. Meanwhile, late flowering, with a prolonged vegetative stage is interesting for increasing fiber yield in low European latitudes (Salentijn et al., 2019). Maintenance of monoecy is essential to increase the homogeneity in dual production of fiber and seeds. This would reduce the efforts to eliminate female and male diecious plants sporadically occurring across generations (Hall et al., 2012; Amaducci et al., 2015; Salentijn et al., 2015). A recent study described large range of variation in flowering time traits and sex determination (monecious/diecious) in a panel of 123 hemp accessions (Petit et al., 2020b). The study described strong influence of genetic components with large heritability estimates for flowering time and sex determination. In addition, significant genotype-by-environment interactions (*G* × *E*) were reported. These interactions indicated significant heritable variation of flowering time sensitive to the environment. Nevertheless, little is known about the genetic and molecular mechanisms that control sex determination, the origin of high plasticity of sex expression, sexual dimorphism and flowering time of hemp. In addition, markers to maintain hemp monoecy or to select for specific flowering time phenotypes are not available.

The present study describes a GWAS approach to characterize the genetic architecture underpinning the length of the vegetative period, flowering time, and sex determination in hemp. A panel of 123 hemp accessions was used to measure these traits in three locations across Europe with contrasting photoperiod regimes. The hemp panel included a large variation of monecious and diecious genotypes and early and late flowering accessions (Petit et al., 2020b). Here, we present QTLs for flowering time traits and sex determination. Furthermore, candidate genes for the QTLs across locations were identified and will contribute to a better understanding of the molecular mechanisms regulating flowering and sex determination in hemp.

## Materials and Methods

### Plant Material

A panel of 123 hemp accessions was used in this study. The panel included a large diversity of accessions from various origins (Europe, China and Canada) and primarily used for different purposes. For specific information of each accession see Supplementary Table 1. The accessions of the panel included large variation in flowering time and in sex type, namely diecious, monecious and accessions with large range of variation in between (Petit et al., 2020b). Plants were grown in three locations across Europe with different photoperiod regimes; in Rovigo (CRA – Centro di ricerca cerealicoltura e colture industriale, Italy, 45°N 11°E), in Chèvrenolles, Neuville-sur-Sarthe (FNPC – Fédération Nationale des Producteurs de Chanvre, France, 48°N 0.2°E), and in Westerlee (VDS – VanDinter Semo BV, Netherlands, 53°N 6°E). Field testing was performed between April and September 2013. Three biological replicates were grown per accession and location in a randomized complete block design and the experimental units were plots of 1m^2^ in CRA and VDS and of 1.5 m^2^ in FNPC. In all three locations, the same sowing density was used to aim a density of 100 plants/m^2^.

### Phenotyping of the GWAS Panel

Each plot was phenotyped for the presence of monecious, independently of the ratio male/female flowers, and diecious plants, independently of the number of male and female plants, according to Petit et al. (2020b). Sex determination was measured per plot assessing “1” when the plants were diecious, “2” when the plants were a mix of diecious and monecious and “3” when the plants were monecious. Data used in the GWAS analyses were provided as the mean between the three pots per accession in each location. Phenotypic values ranged from 1 to 3. Values close to 1 indicated that the accession had mostly diecious plants, values close to 2 indicated that the accession had a mix of diecious and monecious plants and values close to 3 indicated that the accession had mostly monecious plants. The choice of this phenotypic scale was done according to the aim of this research. This study aims to get insights into the sex determination of hemp; monecious (male + female flowers) vs. diecious (male or female flowers), instead of sex expression (male vs. female), as previously done in studies such as Sengbusch (1952) and Faux et al. (2014, 2016).

Flowering time traits were measured in 10 plants of the three middle rows from each plot. Data were provided as the mean per plot. Emergence of the plants was scored in one row per plot at day = N, N + 2, N + 4, N + 7, where N is the day of sowing. Beginning of flowering [FL_Begin in degree-days (the accumulated thermal time over a period with a base temperature of 1°C)] and full flowering (FL_Full in degree-days) were calculated relative to the day of emergence as follows:

(1) FL⁢_⁢Begin=∑CBeginning⁢flowering∘-∑CEmergence,∘

(2) FL⁢_⁢Full=∑CFull⁢flowering∘-∑CEmergence,∘

where Σ°C_Beginning flowering_, Σ°C_Full flowering_, and Σ°C_Emergence_ are the accumulated thermal times, respectively, at the beginning of flowering, at full flowering and at the day of first emergence. The length of vegetative growth period (VEG in days) is the period of vegetative growth of the plants in days, as measured from the day of first emergence until FL_Begin. In diecious plants phenotyping of flowering time was performed in both males and females.

### Genotyping of the GWAS Panel

The genotyping of the GWAS panel was performed using restriction-site associated DNA sequencing (RAD-seq), essentially as previously described by Petit et al. (2020a). DNA was extracted from plants at the juvenile stage before sex was differentiated. Briefly, high quality genomic DNA (2.5 to 5 μg at a concentration ≥25 ng/μl) of the plants was extracted following a modified CTAB method (Petit et al., 2020a). As the sex of the plants was unknown, to cover all allelic variation within accessions and sexes, the genomic DNA of eight randomly selected plants per accessions were pooled in equimolar amounts, resulting in 123 samples. RAD libraries with insert sizes of 300 to 550 bp, were prepared for each sample using the restriction enzyme *Eco*RI, as described by Baird et al. (2008). The 123 samples were paired end sequenced on an Illumina platform (PE150) in two rounds to provide 2 × 1 Gbp genomic data per sample.

After sequencing, quality check adaptors were trimmed from the sequences and low quality reads were removed. Low quality reads comprised reads with >50% of the base quality Q ≤ 12, unknown bases >3%, reads that lack a part of the multiplexing barcode and could not be identified, and reads lacking the key sequence of the enzyme used. Subsequently, clean reads were aligned to the reference *C. sativa* ‘Purple Kush’ assembly (canSat3 version GCA_000230575.1) (van Bakel et al., 2011), using Burrows-Wheeler Alignment Tool (specific BWA parameters: (o) max number or fraction of gap opens = 1; (e) max number or fraction of gap extensions = 50; (m) maximum entries in the queue = 100000) (Li and Durbin, 2009). Picard-tools (v1.118) was used to sort the Sequence Alignment Map (SAM) files by coordinate and convert them to Binary Alignment Map (BAM) files and to mark duplicate reads. The average mapping rate was 55.54% (range 50.3 to 85.7%). Next, SOAPsnp (Li et al., 2009) was used to call SNPs in each sample (SOAP options description: −u (enable rank sum test), −t (set transition/transversion ratio to 2:1 in prior probability); −L [45]; −Q [40]). RAD library preparation, sequencing, SNP calling and genotyping were performed by Beijing Genomics Institute (BGI, Hong Kong).

For each polymorphic site, the allele frequencies (%A, %G, %C, and %T) were calculated per accession and each SNP was scored as the proportion of the major allele per sample. Subsequently, averaged frequencies of major alleles were calculated per SNP over all 123 samples of the GWAS panel. Selection of SNPs for genotyping the panel was performed as follows: 100% call rate in the mapping panel; SNPs with a minor allele frequency below 2% and with a major allele frequency above 98% in the mapping panel were removed; only biallelic SNPs were selected (the frequency sum of the two major alleles in the mapping panel was equal or above 95%); SNPs with a standard deviation in the frequency of the major allele below 0.1 were removed. SNP marker selection was performed in R version 3.4.3 statistical software. In total, 621.452 SNP markers were selected for the genetic analysis (Petit et al., 2020a). The genotypic data of these SNP markers from the GWAS panel were used in the present study. These genotypic data are deposited in the 4TU.ResearchData archive^1^.

### GWAS for Flowering Stages and Sex Determination

In the absence of a complete physical map of the hemp genome, a specific approach was followed for QTL analyses that included: (1) Selection of significant markers by association analyses using a GWAS approach, (2) MultiQTL modeling of significant markers to determine QTLs per trait and per location, and (3) determination of QTLs across locations.

First, a linear mixed model was used to identify significant associations between genotypes and flowering/sex traits (significant QTL-markers), using a kinship correction (VanRaden, 2008) and following the same approach as in Yu et al. (2006); Huang et al. (2010), Malosetti et al. (2011); Zhou and Stephens (2012), Kruijer et al. (2015), and Petit et al. (2020a). The effects of the SNP markers on the phenotypic variation were studied with the fixed effects of the model. The kinship was used to control for population structure effects and was set in the random effects of the model. The kinship was a genomic relationship matrix developed following the method described in VanRaden (2008). Linear mixed model analyses were performed using REML algorithm and the kinship was calculated using R^2^ version 3.4.3 statistical software. The linear mixed model equation of the association with kinship expression is expressed as:

y =X⁢α+K⁢β+e,

where y represents the phenotype, X is the marker, α is the effect size of the marker, K is the kinship for population structure correction, β is the effect of the population structure and e is the residual effect. Xα represents the fixed effects, and Kβ and e the random effects. Wald statistic from the REML analysis was used to assess the significant level of the associations. To account for multiple testing, a Bonferroni correction was performed based on the number of independent markers (Li and Ji, 2005). The cumulative distributions of observed and expected *p*-values were inspected for 3,000 randomly selected SNP markers, to assess the correction for population structure (Yu et al., 2006). The expected *p*-values were the significance level of the associations between genotypes and phenotypes under the assumption that the markers were not associated with the variation of the phenotype. An independent analysis was performed separately for three flowering time traits and sex determination across three locations.

A MultiQTL model was performed on the significant QTL-markers, following a forward selection procedure to identify QTLs associated to a trait, as in Petit et al. (2020a). Here, a QTL was defined as a cluster of significant and collinear QTL-markers represented by the QTL-marker that explains the largest phenotypic variance (representative QTL-marker). The MultiQTL model is the combination of non-collinear QTLs, whereby each QTL explains a specific part of the phenotypic variation in the population. Therefore, first the representative QTL-marker was selected into the model. Then, to avoid multicollinearity between QTLs from the same MultiQTL model, pairwise correlations between the selected QTL-marker and the remaining candidate QTL-markers were assessed. Significant QTL-markers correlated at *r* ≥ 0.3 (∼*r*^2^ ≥ 0.1) were considered collinear to the first one. Significant QTL-markers at *r* < 0.3 (∼*r*^2^ < 0.1) were candidates to add to the model and forward selection was continued with those. The percentage of the total phenotypic variation explained by the genetic effects of the full MultiQTL model was calculated by linear regression (r^2^) between the fitted trait values and the observed trait values.

A Principal Component Analysis (PCA) was performed to graphically represent the results of the GWAS and the multiQTL models. The PCA included all significant QTL-markers from the GWAS and the representative QTL-markers selected in the three MultiQTL models (three locations). The PC1, PC2 and the –log_10_P of the association were plotted in 3D scatter plots to detect the clusters. The 3D scatter plots resembled the peak tops of the QTLs in Manhattan plot distributions.

The three field trials were considered as biological replicates in the absence of data across years. To provide useful QTLs for breeding programs and to further study the genetics of flowering time traits and sex determination across locations, a correlation analysis was performed between the QTL-markers of the three MultiQTL models for each trait. Here, QTLs detected in more than one location were reported. A QTL common across two or three locations was partially composed of the scaffolds with correlated representative QTL-markers (*r* ≥ 0.3).

The threshold to assess multicollinearity between significant markers was based on the relationship pairwise correlation–physical distance between pairs of markers along the largest scaffolds for the *Cannabis sativa* genome (canSat3 version GCA_000230575.1) (van Bakel et al., 2011), as described in Petit et al. (2020a).

Genome-Wide Association Studies, PCA, and correlation analyses were performed in Genstat 19th edition and the 3D scatter plots were performed in Excel version 14.0, using the macro Excel 3D Scatter Plot version 2.1 (Doka, 2013).

### Transcriptome Annotation and Candidate Gene Identification

The representative transcriptome of the drug-type *C. sativa* ‘Purple Kush’ (canSat3) (transcriptome sequencing project: bioproject PRJNA 74271; NCBI GI:351590686 to GI:351629476) was downloaded from the Cannabis Genome Browser Gateway at^3^ (van Bakel et al., 2011). The transcriptome of ‘Purple Kush’ was annotated using Blast2go (Conesa et al., 2005; Conesa and Gotz, 2008; Supplementary File 1). The annotated transcriptomic data are deposited in the 4TU.ResearchData archive^4^. A selection of Purple Kush transcripts with sequence homology (blastx 2.8.0; E value cut of 0.001) to well-known flowering and sex related genes was performed. The selection was based on gene description, gene ontology and relevant research papers on gene functions. Examples of selected genes ontology (GO) terms included P:regulation of photoperiodism, flowering, P:response to red light; P:flower development; P:short-day photoperiodism, flowering; P:photoperiodism; F:photoreceptor activity; F:phosphatidylethanolamine binding; F:DNA photolyase activity; P:positive/negative regulation of gibberellic acid mediated signaling pathway, P:floral organ morphogenesis and P:floral organ development. Finally, genomic scaffolds with significant QTL-markers were analyzed for the presence of the selected transcripts associated to flowering and sex.

### miRNA Analysis

The six microRNA (*csa-miR156*, *csa-miR159a*, *csa-miR171b*, *csa-miR172a*, *csa-miR5021a*, and *csa-miR603*), predicted and validated in *Cannabis sativa* by Das et al. (2015) and Hasan et al. (2016), were subjected to BLASTn (Altschul et al., 1997) search against the genome of *C. sativa* ‘Purple Kush’ assembly (van Bakel et al., 2011). The nucleotide sequences of the miRNA can be found in Table 1.

**TABLE 1:** Nucleotide sequences of 6 miRNA *in silico* predicted and validated in *Cannabis sativa* by Das et al. (2015) and their positions in the cannabis genome (van Bakel et al., 2011).

miRNA	Sequence	Genomic scaffold	Start position	End position
*csa-miR156*	TGACAGAAGAGAGAGAGCAT	scaffold4343	146352	146372
		scaffold123704	2010	2030
*csa-miR159a*	TTTGGATTGAAGGGAGCTCTA	scaffold11004	3	24
*csa-miR171b*	TGATTGAGCCGTGCCAATATC	scaffold900	36428	36449
		scaffold76882	41343	41364
		scaffold113749	6009	6030
*csa-miR172a*	AGAATCTTGATGATGCTGCAT	scaffold140375	1454	1475
*csa-miR5021a*	TGAGAAGAAGAAGAAGAAAA	scaffold5250	22028	22048
		scaffold5438	42938	42958
		scaffold24090	217	237
		scaffold28679	4250	4270
*csa-miR6034*	TCTGATGTATATAGCTTTGGG	−	−	−

## Results

### Phenotypic Variation in Flowering Time and Sex Determination

The extensive phenotypic variation and the large heritable behavior of beginning of flowering, full flowering, length of the vegetative growth period (VEG) and sex determination enabled the study of the genetics underlying flowering and sex in hemp, through a GWAS approach (Supplementary Tables 2, 3; Petit et al., 2020b).

**FIGURE 1:**
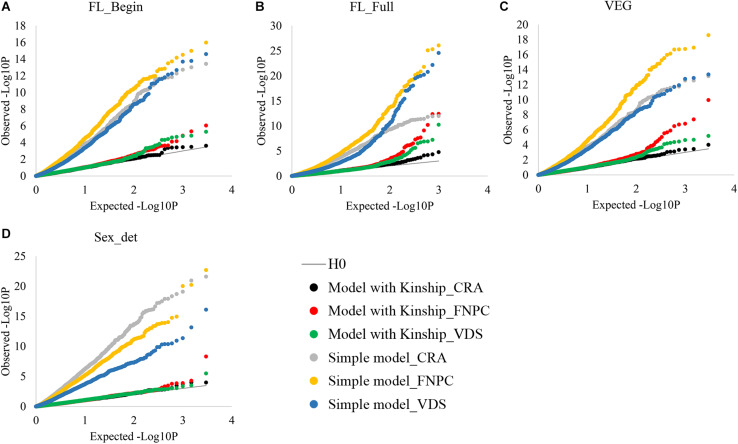
Cumulative plot distributions of beginning of flowering **(A)**, full flowering **(B)**, length of the vegetative growth period (VEG) **(C)**, and sex determination **(D)**. The cumulative distributions of the observed –lop_10_P for the simple model (no corrected for population structure) and the kinship models (corrected for population structure) under the expectation that random SNP markers are unlinked to the polymorphism controlling the traits.

Genome-Wide Association Studies analyses without considering the kinship correction showed large differences between expected and observed *p*-values of the associations for both flowering time and sex determination traits (Figure 1). Yet, when using the kinship correction, observed and expected *p*-values showed similar distributions. Therefore, with the kinship correction, the population structure affecting flowering and sex traits was mostly controlled and allowed the identification of molecular markers associated to the traits.

### Markers and MultiQTL Models for Flowering Stages and Sex Determination

Genome-Wide Association Studies analyses resulted in the identification of 7875 significant associations (−log_10_P ≥ 4.047) between genotypes and phenotypes for the four traits (Supplementary File 2). These associations corresponded to 4225 SNP markers. Among them, 2008 markers were associated to more than one trait and/or location, while 2217 markers were associated to one trait in a single location. Significant markers mapped to single loci on 2195 different genomic scaffolds. The number of significant markers in each genomic scaffold was larger than one in more than 50% of the scaffolds, ranging from 2 to 25 significant markers. The density of significant markers per scaffold was not related to the length of the scaffold sequence. For instance, scaffold 925 harbored 25 significant markers and had a sequence length of ∼42 kbp, while scaffold 13362 also harbored 25 significant markers but with a length of ∼130 kbp. In addition, the distribution of markers within each scaffold showed large variation between scaffolds. For instance, significant markers in scaffold 925 were condensed in a region of ∼18 kbp, while the markers in scaffold 13362 were spread along a larger sequence (∼40 kbp). Furthermore, some significant markers were found close to one edge of the scaffold, such as significant markers from scaffold 13362. This indicated that the flanking genomic sequences of that scaffold can also have significant markers associated to the same trait/s. Therefore, these results suggest that a QTL can span to different genomic scaffolds.

The MultiQTL model analyses identified 63 QTLs (named with the representative QTL-marker) from all MultiQTL models for flowering time and sex determination traits (Table 2). The three MultiQTL models for each trait showed different numbers of QTLs and different explained variances, which are reported in Table 2. The differences between models were likely to be explained by the *G* × *E* interactions affecting flowering and sex traits of hemp (Supplementary Table 3). In addition, models for traits measured in the trials at FNPC and VDS showed similar explained variances. These results suggested similar genetic control of flowering time and sex determination in plants cultivated in FNPC and VDS, while this control might partially differ from that of plants cultivated in CRA.

**TABLE 2:** QTLs for flowering time traits and sex determination of hemp.

Trait	Location	QTLs (n)	Explained variance	Representative QTL-marker
FL_Begin	CRA	2	31.05	scaffold82158_8091; scaffold73915_9581
FL_Begin	FNPC	4	62.01	scaffold3201_6178; scaffold53106_2049; scaffold1534_18526; scaffold26621_95222
FL_Begin	VDS	7	78.31	scaffold2427_91742; scaffold10176_16016; C32110397_2120; scaffold47558_14031; scaffold31281_81403; scaffold21952_1421; scaffold17677_7005
FL_Full	CRA	1	21.9	scaffold15398_26963
FL_Full	FNPC	1	71.05	scaffold112592_6178
FL_Full	VDS	10	88.87	C32042317_287; scaffold15151_24297; scaffold137236_3543; scaffold27165_77253; scaffold4636_30422; scaffold7440_11147; scaffold24246_6032; scaffold4468_47061; scaffold16027_72705; scaffold55664_1689
VEG	CRA	4	39.82	scaffold82158_8091; scaffold2814_39823; scaffold118639_3805; C32101075_450
VEG	FNPC	3	76.45	scaffold27873_3085; scaffold35523_33322; scaffold925_39325
VEG	VDS	9	79.77	scaffold2427_91742; scaffold2696_4663; scaffold33154_78667; scaffold49174_45; scaffold3375_2450; scaffold29523_2165; scaffold63042_7506; scaffold38131_3503; scaffold27843_6598
Sex_det	CRA	2	63.78	scaffold21799_5192; scaffold95298_5540
Sex_det	FNPC	6	81.06	scaffold32801_3642; scaffold9506_1277; scaffold116376_5115; scaffold9283_52650; scaffold3545_18840; scaffold15087_19344
Sex_det	VDS	14	77.5	scaffold46170_31046; scaffold10107_1735; scaffold8222_31139; scaffold7247_5227; scaffold156_223; scaffold23473_1894; scaffold158807_3378; scaffold1968_8392; scaffold41933_1745; scaffold8391_22059; scaffold10301_73345; scaffold40564_1913; scaffold28242_13131; scaffold65111_1444

*The number of QTLs (n, number of groups of collinear markers; at *r* ≥ 0.3) in MultiQTL models generated per trait (FL_Begin, Beginning of flowering; FL_Full, Full flowering; VEG, Length of the vegetative growth period; and Sex_det, Sex determination) and location (CRA, Italy; FNPC, France; and VDS, Netherlands), with the percentage explained phenotypic variance of the full model. Each QTL is named with the marker that explained the largest variance of the QTL (representative QTL-marker). Markers are indicated as the scaffold number and the position of the SNP within the scaffold (canSat3 assembly GAC_000230575.1).*

### QTLs Across Locations for Flowering Time and Sex Traits

**FIGURE 2:**
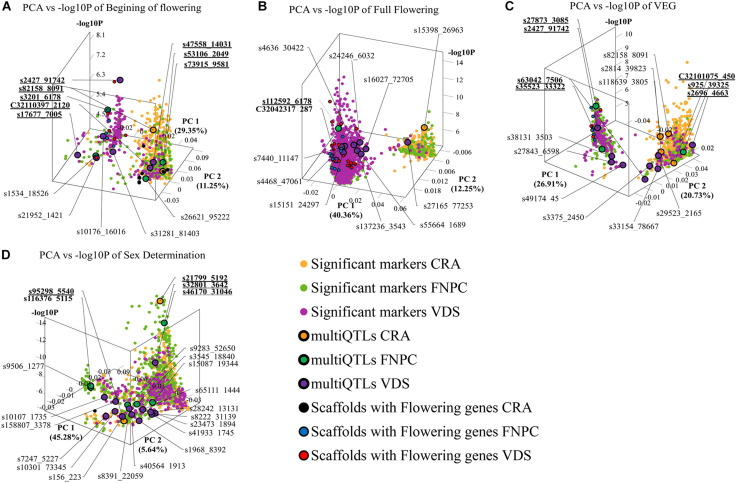
3D scatter plots of PCAs depicting variation in the allele frequency profiles of significant markers for, respectively, beginning of flowering **(A)**, full flowering **(B)**, length of the vegetative growth period (VEG) **(C)**, and sex determination **(D)**. *X*-axes indicate Principal Component 1, *Y*-axes indicate Principal Component 2, and *Z*-axes indicate the significant level of the GWAS association (–log_10_P). Each plot is shown in the angle that represents better the results. Each dot represents a significant QTL-marker from the GWAS analysis. Orange, green, and purple dots indicate significant QTL-markers detected in CRA, FNPC, and VDS, respectively. Orange, green, and purple dots with a black circle represent the representative QTL-markers of QTLs of the MultiQTL models from CRA, FNPC, and VDS, respectively. Black, blue, and red dots with a black circle represent scaffolds with significant QTL-markers associated to the trait in the GWAS with flowering/sex related genes in CRA, FNPC, and VDS, respectively (Table 3). QTL-names in bold and underlined indicate representative QTL-markers from different MultiQTLs models that belong to QTL regions across locations.

**FIGURE 3:**
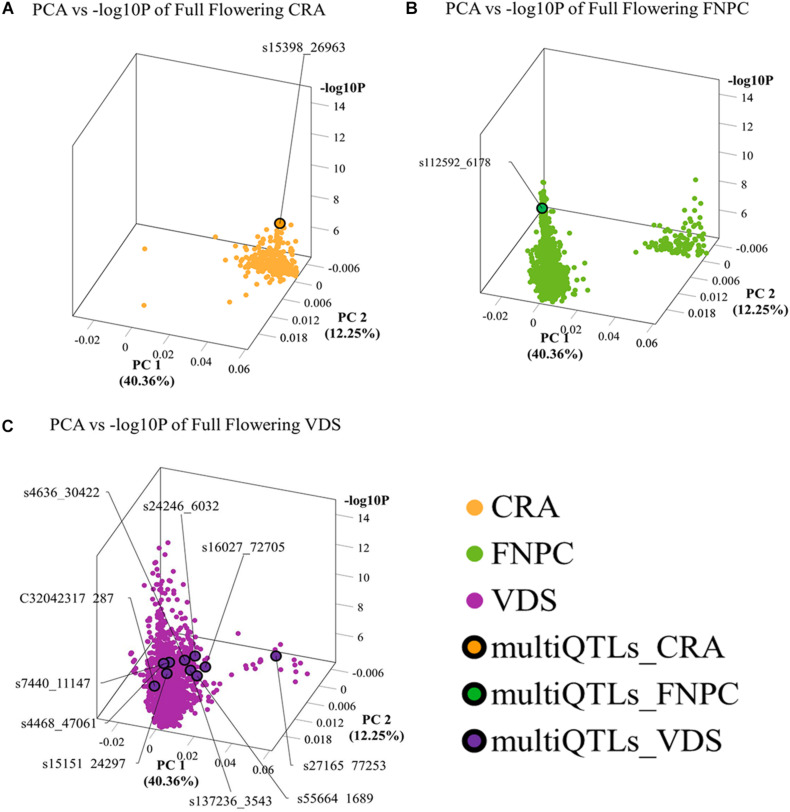
3D scatter plots of PCAs depicting clustering of significant markers on basis of variation in the allele frequency profiles in the mapping population and significance in GWAS, for, respectively, full flowering in CRA **(A)**, full flowering in FNPC **(B)**, and full flowering in VDS **(C)**. *X*-axes indicate Principal Component 1, *Y*-axes indicate Principal Component 2, and *Z*-axes indicate the significant level of the association (–log_10_P). Orange, green, and purple dots indicates the significant markers detected in CRA, FNPC, and VDS, respectively. Orange, green, and purple dots with a black circle represent the representative QTL-markers of the MultiQTL models from CRA, FNPC, and VDS, respectively.

3D scatter plots of the PCAs showed that markers associated to the three flowering time traits clustered in two groups (Figure 2). Yet, the distribution of the markers showed differences across locations. For beginning of flowering (FL_Begin) and length of the vegetative growth (VEG), most markers detected in CRA clustered in a single group, while markers detected in FNPC and VDS clustered in two groups, one of them co-localized with markers detected in CRA. These results indicated that the genetic regulation for both traits (FL_Begin and VEG) is partially common across the three locations. In contrast, for full flowering, most markers from VDS clustered in a single group that only co-localized with markers from FNPC, as shown in Figure 3. Therefore, the results suggested a common genetic regulation of full flowering between FNPC and VDS. The 3D scatter plot from sex determination revealed a single dense cluster of markers from the three locations, alluding to a common genetic regulation of sex determination across all tested locations. Moreover, each cluster included at least one representative QTL-marker from the location (MultiQTL model) where most markers of the cluster were identified.

Finally, correlation analyses between the representative QTL-markers of the three MultiQTL models per trait revealed six QTLs across locations for flowering traits and two for sex determination (Table 3). Among them, four QTLs were identified across two locations, two for length of the vegetative growth (QTL*_*VEG*__2_* and QTL*_*VEG*__3_*) and one, respectively, for full flowering (QTL*_*FL_Full*__1_*) and for sex determination (QTL*_*Sex_det*__2_*). QTL*_*VEG*__2_*, QTL*_*VEG*__3_* and QTL*_*FL_Full*__1_* were identified across FNPC and VDS, while QTL*_*Sex_det*__2_* was identified for both CRA and FNPC locations. Furthermore, four QTLs were identified across three locations, two for beginning for flowering (QTL*_*FL_Beg*__1_* and QTL*_*FL_Beg*__2_*) and one for, respectively, length of the vegetative growth (QTL*_*VEG*__1_*) and sex determination (QTL*_*Sex_det*__1_*).

**TABLE 3:** Identification of QTL regions across locations for flowering time traits and sex determination of hemp.

QTL across locations	Trait	Correlated representative QTL-markers across MultiQTL models		
		CRA	FNPC	VDS
QTL*_*FL_Beg*__1_*	FL_Begin	scaffold82158_8091	scaffold3201_6178	C32110397_2120
				scaffold17677_7005
				scaffold2427_91742
QTL*_*FL_Beg*__2_*	FL_Begin	scaffold73915_9581	scaffold53106_2049	scaffold47558_14031
QTL*_*FL_Full*__1_*	FL_Full	−	scaffold112592_6178	C32042317_287
QTL*_*VEG*__1_*	VEG	C32101075_450	scaffold925_39325	scaffold2696_4663
QTL*_*VEG*__2_*	VEG	−	scaffold27873_3085	scaffold2427_91742
QTL*_*VEG*__3_*	VEG	−	scaffold35523_33322	scaffold63042_7506
QTL*_*Sex_det*__1_*	Sex det	scaffold21799_5192	scaffold32801_3642	scaffold46170_31046
QTL*_*Sex_det*__2_*	Sex det	scaffold95298_5540	scaffold116376_5115	−

*QTLs common across locations were identified by correlation analysis (*r* ≥ 0.3, ∼*r*^2^ ≥ 0.1). QTL across locations, name given to a QTL common across locations; Trait: FL_Begin, Beginning of flowering; FL_Full, Full flowering; VEG, Length of the vegetative growth period; and Sex_det, Sex determination; CRA, representative QTL-markers from MultiQTL models from CRA, Italy; FNPC, representative QTL-markers from MultiQTL models from FNPC, France and VDS, representative QTL-markers from MultiQTL models from VDS, Netherlands.*

### Candidate Genes for Flowering Stages and Sex Determination

In the transcriptome of cannabis ‘Purple Kush’ (van Bakel et al., 2011), 252 transcripts predicted to be involved in flowering were identified (Supplementary Table 4). Among them, 33 transcripts were found in 30 genomic scaffolds with significant QTL-markers collinear to the QTLs across locations (Table 4). Of these 33, 20 candidates were found to be specific for flowering time traits, five specific for sex determination and eight for both type of traits.

**TABLE 4:** Candidate genes in QTL regions across locations.

Scaffold	Markers (n)	Trait/s	QTLs across locations collinear to the markers	Candidate gene (Transcript)	Gene description
scaffold21952	2	FL_BEGIN/VEG	QTL*_*FL_Beg*__1_*/QTL*_*VEG*__2_*	PK17906.1	*Agamous-like* MADS-box AGL93
scaffold16869	4	Sex_det	QTL*_*Sex_det*__1_*	PK04762.1	*Auxin response factor 2B-like isoform X1* (*arf2*)
scaffold21255	1	Sex_det	−	PK19328.1	*Auxin response factor 5* (*arf5*)
scaffold2205	1	FL_FULL	QTL*_*FL_Full*_*	PK10791.1	*Vrn1 gene -* B3 domain-containing transcription factor VRN1-like
scaffold16340	3	Sex_det	QTL*_*Sex_det*__1_*	PK23964.1	bZIP transcription factor 16-like
scaffold2448	4	FL_BEGIN/FLL_FULL/Sex_det/VEG	QTL*_*FL_Beg*__2_*/ QTL*_*VEG*__1_*/QTL*_*Sex_det*__2_*	PK08841.1	bZIP transcription factor 27-like
scaffold11691	3	FL_BEGIN/FL_FULL/VEG	QTL*_*FL_Full*_*	PK19567.1	*Cryptochrome 1 family* (*cry1*)
scaffold31176	3	Sex_det	QTL*_*Sex_det*__1_*	PK09859.1	*Gai* gene - DELLA RGL1-like protein (Repressor of the gibberellin (GA) signaling pathway. Regulates the floral development)
scaffold102797	3	FL_FULL	QTL*_*FL_Full*_*	PK10917.1	*Floricaula leafy* (flower and leaf development)
scaffold19844	3	VEG	QTL*_*VEG*__1_*	PK11476.2	*Flowering locus D (fld)*
scaffold4564	17	FL_BEGIN/FL_FULL/VEG	QTL*_*FL_Beg*__1_*/QTL*_*FL_Full*_*/QTL*_*VEG*__2_*	PK13716.1	Phosphatidylethanolamine-binding PEBP (Regulation of flower development; *Florigen* – *Flowering locus T-like1*)
scaffold3201	4	FL_BEGIN/FL_FULL/VEG/Sex_det	QTL*_*FL_Beg*__1_*/ QTL*_*FL_Full*_*/QTL*_*VEG*__2_*	PK16758.1	*Heading date 3a-like (Flowering locus t-like3)*
scaffold24080	2	FL_FULL	QTL*_*FL_Full*_*	PK08698.1	*Flowering locus T-like4*
scaffold10005	9	FL_FULL/Sex_det	QTL*_*FL_Full*_*/QTL*_*Sex_det*__1_*	PK12609.1	*Flowering time control partial* (Autonomous flowering pathway; inhibiting FLC)
scaffold5190	7	FL_BEGIN/FL_FULL/VEG/Sex_det	QTL*_*FL_Beg*__1_*/ QTL*_*FL_Full*_*/QTL*_*VEG*__2_*	PK04407.1 PK28902.1 PK14825.2	*Gibberellin-20 oxidase Gibberellin 2-beta-dioxygenase 1 Agamous-like* MADS-box AGL6
				PK20658.1	*Probable UDP-N-acetylglucosamine-peptide N-acetylglucosaminyltransferase* SPINDLY (GA balance)
scaffold9737	2	FL_FULL	QTL*_*FL_Full*_*	PK20658.1	*Probable UDP-N-acetylglucosamine-peptide N-acetylglucosaminyltransferase* SPINDLY (GA balance)
scaffold75641	1	FL_FULL	QTL*_*FL_Full*_*	PK05121.1	*Leucine-rich repeat receptor-like serine threonine- kinase* BAM3 isoform X1
scaffold112970	1	VEG	QTL*_*VEG*__1_*	PK15717.1	MADS-box transcription factor (*soc1* gene)
scaffold42686	1	FL_FULL	QTL*_*FL_Full*_*	PK25677.1	*Phytochrome A* (*phyA*)
scaffold33564	1	FL_FULL	QTL*_*FL_Full*_*	PK18424.1	*Phytochrome E* (*phyE*)
scaffold35877	1	sex_det	−	PK12852.1	*Probable lysine-specific demethylase* ELF6 (Circadian clock, photoperiodism, flowering)
scaffold4343	7	FL_BEGIN/FL_FULL/VEG	QTL*_*FL_Beg*__2_*/QTL*_*VEG*__1_*	*csa-miR156* PK22320.1	*miRNA156* with target in *squamosa promoter-binding 1-like* (SPL1-like)
scaffold1953	3	FL_FULL	QTL*_*FL_Full*_*	PK13755.1	*Suppressor of* PHYA-105 1 *(spa1)*
scaffold46774	1	FL_BEGIN/FL_FULL/VEG	QTL*_*FL_Beg*__2_*/QTL*_*VEG*__1_*	PK07299.1	*Ultraviolet-B receptor* (*uvr8*)
scaffold1342	1	FL_FULL	QTL*_*FL_Full*_*	PK10982.1	*Xap5 circadian timekeeper*
scaffold64764	1	FL_FULL	QTL*_*FL_Full*_*	PK05670.1	*Bed* gene – domain-containing RICESLEEPER 2-like
scaffold10189	3	FL_FULL/Sex_det	QTL*_*FL_Full*_*/QTL*_*Sex_det*__2_*	PK10457.1	*Bed* gene – domain-containing RICESLEEPER 2-like
scaffold29022	1	VEG	QTL*_*VEG*__1_*	PK12682.1	*Bed* gene – domain-containing RICESLEEPER 2-like
scaffold12036	3	FL_FULL	QTL*_*FL_Full*_*	PK18183.1	*Zinc finger constans-like 16 (zinc finger constans-like 6)*
scaffold13708	1	FL_FULL	QTL*_*FL_Full*_*	PK23361.1	*Zinc finger constans-like 2*

*Candidate genes located on genomic regions of markers collinear to representative QTL-marker from MultiQTL models (QTL, representative QTL-markers + collinear markers) for flowering time traits (FL_begin, VEG, FL_full) and/or sex determination (Sex_det). Scaffold, genomic scaffold of canSat3 genome that harbors significant markers; Makers (n), number of significant markers present on the scaffold; Candidate gene, transcript of hemp cultivar ‘Purple Kush’ located on a QTL across locations, annotated for a putative function in flowering time/sex determination; Description, description of putative candidate genes. See Supplementary Table 4 for the GenBank accession codes.*

The candidate genes included genes involved in the perception and transduction of light in the flowering pathway. Among them were identified cryptochromes (*cry1*), phytochrome A (*phyA*) and E (*phyE*), suppressor of PHYA-105 *(spa1)*, ultraviolet-B receptors (*uvr8*) and the *xap5* circadian timekeeper gene, which coordinates light signals for proper timing of photomorphogenesis. In addition, several genes that code for transcription factors well-known to regulate flowering were also identified, namely *agamous*, *bed*, *bZIP*, *constans*, *floricaula, leafy*, *flowering locus D*, *flowering locus T*, MAD-box transcription factors, *squamosa* and *vrn1*. Twelve of the above mentioned candidates were detected in scaffolds associated only to QTL*_*FL_FULL*_* for full flowering (Figure 2, Table 4, and Supplementary Tables 4, 5). Furthermore, the regulatory element of flowering genes, *miR156* was found in scaffold 4343 associated to the three flowering time traits (Table 4).

The candidate genes putatively involved in sex determination were identified across ten scaffolds underlying the QTLs. Among them, five scaffolds were also detected in QTLs for flowering traits, sharing some candidate genes. Most of the common candidate genes between flowering and sex determination traits coded for transcription factors, such as *bZIP*, *bed*, *agamous* and *flowering locus T*. The candidate genes specific for sex determination included transcripts related to the metabolism and regulation of phytohormones, such as *auxin response factors* genes (*arf2* and *arf5*) and *gibberellin acid insensitive* gene (*gai* gene – DELLA proteins). The candidate genes specific for sex determination were mostly identified in QTL*_*Sex_det*__1_* across the three environments (Figure 2, Table 4, and Supplementary Tables 1, 2).

## Discussion

Flowering time and sex determination of *Cannabis sativa* L. strongly influence fiber quality and seed yield (Mandolino and Carboni, 2004; Amaducci and Gusovius, 2010; Faux et al., 2013; Amaducci et al., 2015; Liu et al., 2015; Salentijn et al., 2015, 2019; Petit et al., 2020b). Flowering triggers the secondary bast fiber formation and an intense lignification of the fibers to prepare the plant to hold the heavy buds loaded with seeds (Westerhuis et al., 2019). This natural process is associated with a drop in fiber quality. This is because secondary bast fibers and lignin provide stiffness to the fibers and make them coarse (van Dam, 2008). Monecious cultivars are associated with uniformity in fiber and seed yield and quality (Mandolino and Carboni, 2004; Salentijn et al., 2015). Thus, understanding the genetic mechanisms underlying such complex traits will help to develop molecular markers for breeding for different type of hemp cultivars, such as for fiber and oil production. Molecular markers associated with flowering time traits will contribute to develop early or late flowering hemp cultivars better adapted to specific photoperiod regimes with desired seed and/or fiber yields and qualities. For instance, late flowering cultivars will have a prolonged vegetative stage and will increase fiber yield and quality in low European latitudes (Salentijn et al., 2019). Molecular markers associated with monoecy will greatly contribute to improve monecious accessions with maintained sex determination across generations (more stable monoecy). This would reduce the efforts to genotype all plants with the already existing male specific molecular marker (Moliterni et al., 2004) at early development plant stages to eliminate diecious plants spontaneously occurring across generations (Hall et al., 2012; Amaducci et al., 2015; Salentijn et al., 2015). Consequently, increased stable monoecy will increase the uniformity of the fiber and seed production, with positive effects on the quality of end-products.

Here, association studies were performed to identify molecular markers and QTLs for flowering time and sex determination using a panel of 123 hemp accessions. The phenotyping was performed in three locations with contrasting environments across Europe. The genomic regions associated with the QTLs were further characterized to identify putative candidate genes explaining the molecular basis of these traits in hemp.

### Elucidating Molecular Markers and QTLs for Hemp Flowering Time and Sex Determination

In this study, more than 4000 molecular markers were found significantly associated (−log_10_P ≥ 4.047) to the phenotypic differences of flowering and sex traits from the hemp panel. The extensive and heritable variation of the flowering and sex traits across different environments (*H*^2^ = 0.93–0.95) (Supplementary Table 3) made possible to study the genetics of these complex traits in hemp (Petit et al., 2020b). In addition, the genomic scaffolds with significant markers condensed on one edge of the scaffold indicate that a QTL can be composed of several genomic scaffolds that would flank in a complete genome sequence (Petit et al., 2020a). Correlation analyses between significant markers identified 63 QTLs from all MultiQTL models for flowering time and sex determination traits (Table 2). The three MultiQTL models (three locations trials) for each trait showed different number of QTLs and explained phenotypic variance. In addition, the study to identify common QTLs across locations detected only six QTLs across two or three locations for flowering time traits and two for sex determination (Table 3). These results are likely to be explained by the relatively large genotype-by-environment (*GxE)* interactions affecting these traits (Supplementary Table 3; Petit et al., 2020b). The *GxE* interactions represent the fraction of the phenotypic variation that is heritable but sensitive to the environment. Flowering is an essential biological process for many plants as the survival of the species depends on it (Mouradov et al., 2002). The beginning of flowering is characterized by the activation of floral meristem identity genes. These genes can be triggered by different flowering pathways, such as photoperiod dependent pathways, temperature (vernalization) dependent pathways, autonomous dependent pathways and phytohormones (GA) dependent pathways [reviewed in Salentijn et al. (2019)]. As flowering and sex determination of hemp are known to be affected by the environment (Truta et al., 2007; Amaducci et al., 2008a, 2012; Hall et al., 2014; Sawler et al., 2015; Salentijn et al., 2015, 2019; Petit et al., 2020b) and many genes are involved in regulation of flowering time [reviewed in Salentijn et al. (2019)], common QTLs across locations represent the common genetic basis of flowering and sex determination that shows low sensitivity to the environment. Meanwhile, QTLs identified in a single specific environment could represent a fraction of the heritable variation that is more affected by the environment. Therefore, the variation associated to these QTLs might have an effect to flowering or sex determination only under specific environmental conditions. However, this hypothesis needs to be supported with technical replicates of each location trial across years. These data would allow to study the behavior of the QTLs toward the environment.

### Elucidating Molecular Mechanisms in Hemp Flowering Time

**FIGURE 4:**
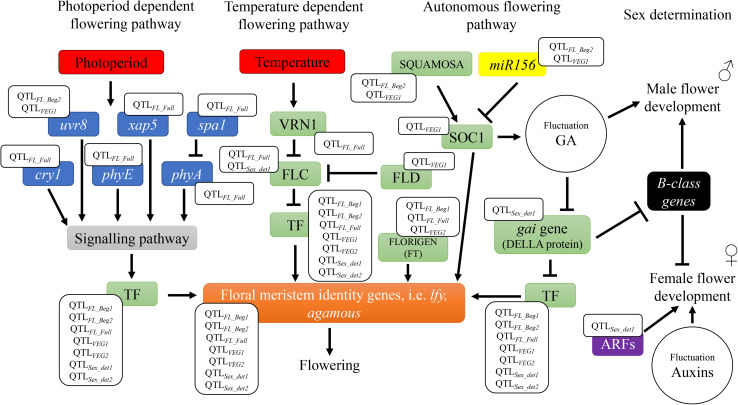
Flowering and sex determination metabolic pathways: identification of candidate genes underneath the QTLs. The QTLs for flowering time are found in different flowering dependent pathways (photoperiod, temperature, and endogenous flowering pathways). Photoperiod pathway involves genes of the perception and transduction of light signals [ultraviolet-B receptors (*uvr8*), circadian timekeeper (*xap5*), suppressor of PHYA-105 (*spa1*), cryptochromes (*cry1*), phytochrome A (*phyA*), and phytochrome E (*phyE*)]. Temperature pathway involves *vrn1*, a vernalization dependent transcription factor. Both photoperiod and temperature pathways activate signaling pathways and/or transcription factors involved in the endogenous flowering pathway to regulate floral meristem identity genes, such as *leafy* (*lfy*). Genes that code for these transcription factors include *flowering locus C* (FLC), *flowering locus D* (FLD), *flowering locus T* (FLORIGEN or FT), s*uppressor of overexpression of constans1* (*soc1*), and *gibberellic acid insensitive* (*gai* gene – DELLA protein), among others. TF is used to summarize all transcription factors inducing floral meristem identity genes. Endogenous pathway also include the regulatory element of flowering genes, *miR156*. The QTLs for sex determination are found in metabolic pathways involved in regulation of phytohormones gibberellic acid (GA) or auxins. These pathways include *B-class* homeotic genes involved in the development of male flower organs and *auxin response factors* genes (ARFs) involved in female flower development.

The six QTLs across locations and the 28 candidate genes underpinning these genetic markers, included key regulators of hemp flowering time involved in different flowering pathways, as depicted in Figure 4. Four candidate genes involved in the perception and transduction of environmental signals (photoperiod flowering pathway), such as *cryptochrome 1* (*cry1*), *phytochrome A* (*phyA*) and *E* (*phyE*), and *suppressor of phytochrome A* (*spa1*) were identified in the QTL*_*FL_FULL*_*, for full flowering, across two environments. These genes code for photoreceptors that regulate light responses under different light conditions, such as light quantity, quality and timing (e.g., *cry1*, *phyA*, and *phyE*) and for proteins that inhibit photoreceptor’s activity (e.g., *spa1*) (Lin, 2000). The activity of these genes triggers or inhibits a signaling pathway that regulates flowering (Lin, 2000; Figure 4). Previous research in arabidopsis and tobacco concluded that mutations in cryptochromes [*cry1* (Lin et al., 1995) and c*ry2* (Guo et al., 1998)] and phytochromes [*phyA* (Johnson et al., 1994; Reed et al., 1994)] result in alterations in the flowering time. QTL*_*FL_FULL*_* was identified across FNPC and VDS, the two Northern European locations of the present study with a longer long-day photoperiod regime compared to CRA. The differences in photoperiod regimes across locations and the role of the photoreceptors and their suppressors contribute to understand the function of this QTL in controlling flowering time. The expression of the arabidopsis *cry1* gene in transgenic tobacco has been reported to increase the hypersensitivity to blue, UV-A and green light (Lin et al., 1995). Therefore, the quantitative genetic variation of QTL*_*FL_FULL*_* in hemp can affect the function of the genes and generate a hypersensitivity to specific light conditions (e.g., specific environment from Northern Europe), triggering a response in flowering time.

Furthermore, the candidate gene *vrn1*, also identified in QTL*_*FL_FULL*,_* can provide deeper insights into flowering time of hemp. The gene *vrn1* is a vernalization related gene identified in arabidopsis (Michaels and Amasino, 1999; Levy et al., 2002; Bouché et al., 2016; Cheng et al., 2017). This gene codes for a protein that functions in stable repression of the major actor in the vernalization pathway: the floral repressor transcription factor, FLOWERING LOCUS C (FLC). FLC represses the expression of flowering integrator genes, thereby inhibiting the plants to flower (Figure 4). A period of low temperatures is needed to trigger the expression of *vrn1*, which in turn leads to flowering (Cheng et al., 2017). Hemp has no vernalization requirements but temperature is known to be a factor affecting the length of the juvenile stage. Low temperatures increase the duration to flowering (Lisson et al., 2000; Amaducci et al., 2012; Salentijn et al., 2019). It is known that flowering genes can have specific function in different plants species [reviewed in Salentijn et al. (2019)]. As hemp has no vernalization requirements, quantitative genetic variation in *vrn1* in association to temperature sensitivity could have a specific function in hemp in controlling the length of the juvenile stage and consequently flowering time. This candidate gene is of great significance for further studies, such as candidate gene functional approaches where this gene is mutated, to study the specific effect of *vrn1* in hemp and its putative consequences for flowering.

Genes coding for transcription factors involved in flowering, such as *agamous*, *bZIP*, *soc1* (MADS-box transcription factor), *flowering locus D*, *flowering locus T* and *squamosa* and the flowering regulatory element, *miRNA156* were identified in QTLs for beginning of flowering and length of vegetative growth. All these genes are involved in autonomous flowering pathways, which operate independently from the photoperiod and temperature pathways (Figure 4). For instance, *squamosa* and *miRNA156* are involved in the regulation of the flowering gene *suppressor of overexpression of constans1* (*soc1*) (Wu et al., 2009; Hyun et al., 2016). *Squamosa* gene codes for a transcription factor that promotes the expression of *soc1*, while *miR156* represses its expression. In arabidopsis, *soc1* codes for a MADS-box transcription factor that can directly activate the floral meristem identity gene *leafy*, which directly triggers the flowering meristems of the plants. In addition, *soc1* can generate fluctuations in the balance of the phytohormone gibberellic acid (GA) and activate flowering (Lee and Lee, 2010; Immink et al., 2012; Hyun et al., 2016). *Flowering locus T*, also known as *ft* or *florigen*, is a mobile flower-promoting signal transported from the leaves to the meristems elsewhere in the plant. *Flowering locus T* induces the expression of other flowering identity genes (e.g., *agamous*), which induce meristems to flower. *Flowering locus D* codes for another transcription factor (FLD) that, as *vrn1* gene, controls the floral repressor FLC. However, the mode of action of FLD is independent of the temperature. This transcription factor probably mediates histone demethylation at FLC locus, inactivating the expression of FLC and consequently FLD triggers flowering (He et al., 2003; Jiang et al., 2007; Liu et al., 2007). Therefore, genetic variation in these genes reported to affect flowering, through autonomous flowering pathways, can lead to flowering with less sensitivity to the environment.

### Molecular Mechanisms in Hemp Sex Determination

The identification of the two QTLs for sex determination across locations is a significant step toward understanding the sex determination system (monecious and diecious) of cannabis. Two auxin response factor genes (*arf2* and *arf5*), *bZIP* transcription factor 16-like and gene *gibberellic acid insensitive* (*gai*) that codes for the DELLA RGL1-like protein were identified in QTL*_*Sex_det*__1_* for sex determination. These genes are involved in the balance of the phytohormones auxins and gibberellic acid, which are known to play an active role in the sex expression (male or female) in many crops, such as hemp or spinach [(Heslop-Harrison, 1956; Chailakhyan and Khryanin, 1978; Galoch, 2015) and reviewed in Salentijn et al. (2019)]. For instance, in spinach the *gibberellic acid insensitive* (*Spgai*) gene was two-fold higher expressed in inflorescences from females compared to male inflorescences (West and Golenberg, 2018). Both, the exogenous GA application and the reduction of *Spgai* gene expression in female plants, induced the development of male flowers in spinach (Figure 4). Based on their research they proposed a model for the sex determination of monecious plants in spinach, named M locus, which could apply to hemp. In conditions of high expression of *Spgai* there was an inhibition of the spinach *B-class* homeotic genes. These *B-class* homeotic genes are masculinizing factors of flower development, promoting the formation of male organs and at the same time they inhibit female organ formation in flowers (Figure 4). When applying GA to spinach, *Spgai* gene was inhibited and thus the inhibition on the masculinizing *B-class* genes was released, resulting in the formation of male organs. Soldatova and Khryanin (2010) also reported a masculinization effect of female hemp plants owed to external treatment with GA.

Furthermore, Heslop-Harrison (1956) provided evidences that the treatment of male diecious hemp with auxins showed the development of female flowers (Figure 4). In addition, expression analyses from floral meristems to study the differences in mRNA between male and female diecious hemp showed that mRNA involved in auxins were overexpressed in female plants (Moliterni et al., 2004). Auxins are phytohormones known to be involved in flower development (Hardtke and Berleth, 1998). Auxins regulate this process by controlling gene expression via transcription factors AUXIN RESPONSE FACTORS (ARF). These transcription factors control the expression of genes involved in female flower development (Li et al., 2016; Figure 4). For example, the transcription factor ARF8 acts as an inhibitor to stop further development of carpel in the absence of fertilization and the generation of signals to induce seed development (Goetz et al., 2006). Arabidopsis mutant for the *auxin response factor 8* (*arf8*) gene developed seedless fruits (Goetz et al., 2006). In addition, Liu et al. (2014) reported floral development defects and female sterility in tomato where *arf6* and *arf8* genes were down-regulated with microRNAs.

It is likely that QTL*_*Sex_det*__1_* contributes to regulate sex determination by controlling the expression of male and female inflorescences in a female genetic background, for example through downregulation of *gai* gene and/or downregulation of *auxin response factor* genes. Both effects might promote the development of male flower organs in female plants, leading to a monecious hemp. This is essentially because the positive effect alleles of QTL*_*Sex_det*__1_* are associated to promote monoecy in hemp plants, while the negative effect alleles to promote dioecy (phenotypic values close to 3 indicate monoecy and values close to 1 dioecy, according to the phenotypic scale used in this study). The common genetic basis of monoecy and dioecy sex determination of hemp are in accordance to the common markers between monecious and diecious populations reported by Faux et al. (2016). The argumentation of downregulation of *auxin response factor* genes is also in agreement with Moliterni et al. (2004). They suggested that the repression of female characteristics implies the down-regulation of the genes involved in pathways more strictly regulated to the differentiation of the female sex.

Previous studies reported that monecious hemp plants carry the homogametic sex chromosomes XX (Faux et al., 2014) and QTLs associated to sex expression (male vs. female diecious plants and ratio male/female inflorescences in monecious plants) were located on the sex chromosomes, based on genetic maps (Faux et al., 2016). However, the lack of a complete genome sequence do not allow to map the QTL*_*Sex_det*__1_* in any specific chromosome. Thus, QTL*_*Sex_det*__1_* can be either located on the X chromosome, as some sex QTLs from Faux et al., 2016, or in any of the autosomes. Finally, molecular markers composing this QTL can be used as markers to directly select for monecious plants, differently than the male specific SCAR marker already available. This is because the male specific marker only allows to discriminate between male and female plants (Moliterni et al., 2004).

## Conclusion

The results of this study prescribe new prospects to understand the genetics basis of flowering time and sex determination in hemp. Molecular SNP markers and QTLs were identified for these quantitative traits. Genes involved in the photoperiod and temperature flowering pathways, such as genes involved in the perception and transduction of environmental signals (i.e., light), and genes involved in the autonomous and phytohormones flowering pathways, such as flowering transcription factors, were identified in QTLs for flowering time. About sex determination, genes involved in regulating the balance of phytohormones gibberellic acid (GA) and auxins were identified. The alleles with positive effects of these sex QTLs were found to promote monecious phenotypes. Finally, the SNP markers composing the QTLs can be used to develop new hemp cultivars with early or late flowering time behaviors and to select for monecious plants. SNP markers associated with sex determination will increase the stability of monoecy determination in monecious hemp cultivars.

## Data Availability Statement

The datasets presented in this study can be found in online repositories. RADseq data, selected informative SNP markers and the annotated transcriptomic data of the marihuana cultivar C. sativa ‘Purple Kush’ are deposited in the 4TU.ResearchData archive (doi: 10.4121/12826832 and doi: 10.4121/13121885.v1).

## Author Contributions

JP designed and performed the experiments, analyzed the data, and wrote the manuscript. ES helped designing and performing the experiments, helped analyzing data, and revised the manuscript. M-JP helped analyzing the data and revised the manuscript. CD helped designing and performing the experiments and revised the manuscript. LT coordinated and supervised this study, delineated the experimental strategy, discussion of the outcomes, and revised the manuscript. All authors contributed to the article and approved the submitted version.

## Conflict of Interest

The authors declare that the research was conducted in the absence of any commercial or financial relationships that could be construed as a potential conflict of interest.

## Footnotes

1 https://doi.org/10.4121/12826832

2 http://www.r-project.org/

3 http://genome.ccbr.utoronto.ca/cgi-bin/hgGateway

4 https://doi.org/10.4121/13121885.v1
