# Supplementary material for: Genetic Architecture of Flowering Time and Sex Determination in Hemp (Cannabis sativa L.): A Genome-Wide Association Study
Source: Front Plant Sci. 2020 Nov 4;11:569958. doi: 10.3389/fpls.2020.569958 (PMC7672029; doi:10.3389/fpls.2020.569958)
Supplement: Supplementary file 1 [file Data_Sheet_1.docx]

**Supplementary Table 1.** Panel of 123 hemp (*Cannabis sativa* L.) accessions from (Petit et al., 2020). Population types B, L and W stand for breeding material, landraces and wild material, respectively. Accession type are based on use.

| MultiHemp code | Accession name / Code | Origin | Accession type | Population type | Provider |
| --- | --- | --- | --- | --- | --- |
| MH-AGM-701 | Fibrol / - | Hungary | Fibre | B | AGM |
| MH-AGM-702 | Tiborszallasi / - | Hungary | Fibre | B | AGM |
| MH-AGM-703 | Tisza / - | Hungary | Fibre | B | AGM |
| MH-AGM-704 | KC Dora / - | Hungary | Fibre | B | AGM |
| MH-AGM-705 | Monoica / - | Hungary | Fibre | B | AGM |
| MH-CAAS-601 | CYM171 / - | China | Fibre | B | CAAS |
| MH-CAAS-602 | CYM28 / - | China | Fibre | B | CAAS |
| MH-CAAS-603 | Yunma 5 / - | China | Fibre | B | CAAS |
| MH-CAAS-604 | CYM49 / - | China | Fibre | B | CAAS |
| MH-CAAS-605 | CYM273 / - | China | Fibre | B | CAAS |
| MH-CRA-401 | CRA_1 / - | Italy | Fibre | B | CRA |
| MH-CRA-402 | CRA_2 / - | Italy | Fibre | B | CRA |
| MH-CRA-404 | Delta llosa / - | Spain | Fibre | B | CRA |
| MH-CRA-405 | CRA_4 / - | Italy | Fibre | B | CRA |
| MH-CRA-406 | Carma Monoica / - | Italy | Fibre | B | CRA |
| MH-CRA-407 | Supermono / - | Italy | Fibre | B | CRA |
| MH-CRA-408 | Fibranova (CRA_5) / - | Italy | Fibre | B | CRA |
| MH-CRA-409 | Carmagnola / - | Italy | Fibre | B | CRA |
| MH-CRA-410 | Ermes A / - | Italy | Fibre | B | CRA |
| MH-CRA-411 | CS (CRA_6) / - | Italy | Fibre | B | CRA |
| MH-CRA-412 | Carmaleonte / - | Italy | Fibre | B | CRA |
| MH-CRA-413 | CRA_7 / - | Italy | Fibre | B | CRA |
| MH-CRA-414 | W-1 / - | Italy | Fibre | B | CRA |
| MH-CRA-415 | Zenit / - | Romania | Fibre | B | CRA |
| MH-CRA-416 | Denise / - | Romania | Fibre | B | CRA |
| MH-CRA-417 | CRA_8 / - | Italy | Fibre | B | CRA |
| MH-CRA-418 | SVGB-10611 / - | Italy | Fibre | B | CRA |
| MH-CRA-419 | USO 14 Monoica / - | Ukraine | Fibre | B | CRA |
| MH-CRA-420 | USO 31 / - | Ukraine | Fibre | B | CRA |
| MH-FNPC-201 | - / A11-121-1 | France | Fibre | B | FNPC |
| MH-FNPC-202 | - / A11-121-2 | France | Fibre | B | FNPC |
| MH-FNPC-203 | - / A11-121-3 | France | Fibre | B | FNPC |
| MH-FNPC-204 | - / A11-121-4 | France | Fibre | B | FNPC |
| MH-FNPC-205 | - / A11-121-5 | France | Fibre | B | FNPC |
| MH-FNPC-206 | - / A11-121-6 | France | Fibre | B | FNPC |
| MH-FNPC-207 | - / A11-121-7 | France | Fibre | B | FNPC |
| MH-FNPC-209 | - / A11-121-9 | France | Fibre | B | FNPC |
| MH-FNPC-210 | - / A11-121-10 | France | Fibre | B | FNPC |
| MH-FNPC-211 | - / A11-121-11 | France | Fibre | B | FNPC |
| MH-FNPC-212 | - / A11-121-12 | France | Fibre | B | FNPC |
| MH-FNPC-213 | - / A11-121-13 | France | Fibre | B | FNPC |
| MH-FNPC-214 | - / A11-121-14 | France | Fibre | B | FNPC |
| MH-FNPC-215 | - / A11-121-15 | France | Fibre | B | FNPC |
| MH-FNPC-216 | - / A11-121-16 | France | Fibre | B | FNPC |
| MH-FNPC-217 | - / A11-121-17 | France | Fibre | B | FNPC |
| MH-FNPC-218 | - / A11-121-18 | France | Fibre | B | FNPC |
| MH-FNPC-219 | - / A11-121-19 | France | Fibre | B | FNPC |
| MH-FNPC-220 | - / A11-121-20 | France | Fibre | B | FNPC |
| MH-FNPC-221 | - / A11-121-21 | France | Fibre | B | FNPC |
| MH-FNPC-222 | - / A11-121-22 | France | Fibre | B | FNPC |
| MH-FNPC-223 | - / A11-121-23 | France | Fibre | B | FNPC |
| MH-FNPC-224 | - / A11-121-24 | France | Fibre | B | FNPC |
| MH-FNPC-225 | - / A10-122-1 | France | Fibre | B | FNPC |
| MH-FNPC-226 | - / A10-122-2 | France | Fibre | B | FNPC |
| MH-FNPC-227 | - / A10-122-4 | France | Fibre | B | FNPC |
| MH-FNPC-228 | - / A103-122-1 | France | Fibre | B | FNPC |
| MH-FNPC-229 | - / A103-122-2 | France | Fibre | B | FNPC |
| MH-FNPC-230 | - / A103-122-3 | France | Fibre | B | FNPC |
| MH-FNPC-231 | - / A103-122-4 | France | Fibre | B | FNPC |
| MH-FNPC-232 | - / A103-122-6 | France | Fibre | B | FNPC |
| MH-FNPC-233 | - / A103-122-8 | France | Fibre | B | FNPC |
| MH-FNPC-234 | - / A103-122-10 | France | Fibre | B | FNPC |
| MH-FNPC-235 | - / A9-122-1 | France | Fibre | B | FNPC |
| MH-FNPC-236 | - / A9-122-2 | France | Fibre | B | FNPC |
| MH-FNPC-237 | - / A9-122-3 | France | Fibre | B | FNPC |
| MH-FNPC-238 | - / A9-122-4 | France | Fibre | B | FNPC |
| MH-FNPC-239 | - / A102-122-1 | France | Fibre | B | FNPC |
| MH-FNPC-240 | - / A102-122-2 | France | Fibre | B | FNPC |
| MH-FNPC-241 | - / A102-122-3 | France | Fibre | B | FNPC |
| MH-FNPC-242 | - / A102-122-4 | France | Fibre | B | FNPC |
| MH-FNPC-243 | - / A102-111-1 | France | Fibre | B | FNPC |
| MH-FNPC-244 | - / A102-111-2 | France | Fibre | B | FNPC |
| MH-FNPC-245 | - / A7-104-1 | France | Fibre | B | FNPC |
| MH-FNPC-246 | - / A7-105-4 | France | Fibre | B | FNPC |
| MH-FNPC-248 | - / B6-093-3 | France | Fibre | B | FNPC |
| MH-FNPC-250 | - / B6-093-17 | France | Fibre | B | FNPC |
| MH-FNPC-251 | Férimon / - | France | Fibre | B | FNPC |
| MH-FNPC-252 | Fédora 17 / - | France | Fibre | B | FNPC |
| MH-FNPC-253 | Félina 32 / - | France | Fibre | B | FNPC |
| MH-FNPC-254 | Epsilon 68 / - | France | Fibre | B | FNPC |
| MH-FNPC-255 | Futura 75 / - | France | Fibre | B | FNPC |
| MH-FNPC-256 | Santhica 27 / - | France | Fibre | B | FNPC |
| MH-IWNRZ-901 | Bialobrzeskie / - | Poland | Fibre | B | IWNRZ |
| MH-IWNRZ-902 | Beniko / - | Poland | Fibre | B | IWNRZ |
| MH-IWNRZ-903 | Tygra / - | Poland | Fibre | B | IWNRZ |
| MH-LARC-501 | Katlakalna / - | Latvia | Fibre | B | LARC |
| MH-UOY-801 | Finola / - | Finland | Seed | B | UOY |
| MH-VDS-301 | Chameleon / - | Netherlands | Fibre | B | VDS |
| MH-VDS-302 | Marcello / - | Netherlands | Fibre | B | VDS |
| MH-VDS-303 | Markant / - | Netherlands | Fibre | B | VDS |
| MH-VDS-304 | Ivory / - | Netherlands | Fibre | B | VDS |
| MH-WU-101 | JSO 16 / 891229 | Russia | Fibre | B | WUR |
| MH-WU-102 | Ajkai-A-TF / 891054 | Hungary | Fibre | L | WUR |
| MH-WU-103 | Fibrimon 56 / 880828 | France | Fibre | B | WUR |
| MH-WU-104 | Rastislavicke / 880816 | Slovakia | Fibre | B | WUR |
| MH-WU-105 | Krasnodarskaja 56 / 891333 | Ukraine | Fibre | B | WUR |
| MH-WU-106 | Dneprovskaja 84 / 921054 | Russia | Fibre | L | WUR |
| MH-WU-107 | - / 883290 | Russia | Fibre | L | WUR |
| MH-WU-108 | Lovrin 110 / 883173 | Romania | Fibre | B | WUR |
| MH-WU-109 | Bialobrzeskie / 891223 | Poland | Fibre | B | WUR |
| MH-WU-110 | - / 880973 | Spain | - | - | WUR |
| MH-WU-111 | Kompolti Sargászáru / 883049 | Hungary | Fibre | B | WUR |
| MH-WU-112 | - / 883262 | Spain | - | - | WUR |
| MH-WU-113 | Kompolti hybrid TC / 891070 | Hungary | Fibre | B | WUR |
| MH-WU-114 | Fibrimon 56 / 891158 | France | Fibre | B | WUR |
| MH-WU-115 | - / 921203 | Canada | - | W | WUR |
| MH-WU-116 | Panorama var. globosa / 910914 | Hungary | Ornamental | B | WUR |
| MH-WU-117 | Silistrenski / 901107 | Bulgaria | Fibre | B | WUR |
| MH-WU-118 | Csehslovák-A-TF / 891068 | Slovakia | Fibre | - | WUR |
| MH-WU-119 | - / 891288 | Poland | Fibre | - | WUR |
| MH-WU-120 | - / 891090 | Turkey | - | L | WUR |
| MH-WU-121 | Komoroi-A-TF / 891046 | Hungary | Fibre | L | WUR |
| MH-WU-122 | - / 883289 | Russia | Fibre | L | WUR |
| MH-WU-123 | Juznaja Odnovremenno / 883293 | Russia | Fibre | B | WUR |
| MH-WU-124 | - / 891240 | Spain | - | - | WUR |
| MH-WU-125 | Orosi-A-TF / 891059 | Hungary | Fibre | - | WUR |
| MH-WU-126 | Kompolti / 883048 | Hungary | Fibre | - | WUR |
| MH-WU-127 | Dneprovskaja odnodomnaja 6 / 891326 | Ukraine | Fibre | B | WUR |
| MH-WU-128 | - / 891327 | - | Fibre | L | WUR |
| MH-WU-129 | Superfibra / 883040 | Italy | Fibre | B | WUR |
| MH-WU-130 | - / 891057 | Hungary | - | L | WUR |
| MH-WU-131 | - / 891094 | Turkey | - | L | WUR |
| MH-WU-132 | - / 880817 | Germany | - | - | WUR |

**Supplementary Table 2.** Genetic variability in flowering time and sex determination traits of hemp grown in three contrasting locations. Means, standard deviations and coefficient of variation (CV%). Data adapted from (Petit et al., 2020).

| Trait | Abbreviation | Location | | | | | |
| --- | --- | --- | --- | --- | --- | --- | --- |
|  |  | CRA | | FNPC | | VDS | |
|  |  | Mean ± SD | CV% | Mean ± SD | CV% | Mean ± SD | CV% |
| Beginning of flowering time (∑˚C) | FL_BEGIN | 1055.22 ± 317.81 | 30.12 | 1233.18 ± 342.12 | 27.74 | 1247.86 ± 321.92 | 25.8 |
| Full flowering time (∑˚C) | FL_FULL | 1676.03 ± 521.75 | 31.13 | 1541.52 ± 437.34 | 28.37 | 1432.41 ± 502.09 | 35.05 |
| Begin of flowering relative to day of first emergence (days) | VEG | 57.23 ± 14.89 | 26.01 | 65.85 ± 17.41 | 26.43 | 85.93 ± 17.6 | 20.48 |
| Sex determination | Sex_det | 2.26 ± 0.89 | 39.59 | 2.31 ± 0.95 | 41.13 | 2.1 ± 0.7 | 33.59 |

CV% = $\frac{Standard deviation}{Mean}*100$.

**Supplementary Table 3.** Variance components and broad-sense heritability (H^2^) of three flowering time traits and sex determinism. The variances explained by each component are shown as the proportion of total variance (%). Data adapted from (Petit et al., 2020).

| Trait | Abbreviation | Location (*L*%) | Block within Location (*B*%) | Genotype (*G*%) | Genotype x Location (*LG*%) | Error (*ε*%) | H^2^ |
| --- | --- | --- | --- | --- | --- | --- | --- |
| Beginning flowering time (∑˚C) | FL_BEGIN | 9.06 | 0.4 | 74.43 | 8.97 | 7.15 | 0.95 |
| Full flowering time (∑˚C) | FL_FULL | 4.62 | 0.05 | 78.86 | 14.66 | 1.81 | 0.94 |
| Begin of flowering relative to day of first emergence (days) | VEG | 42.44 | 0.3 | 46.13 | 6.16 | 4.98 | 0.95 |
| Sex determinism | Sex_det | 2.53 | 0.91 | 68.9 | 10.52 | 17.13 | 0.93 |

**Supplementary Table 4.** List of flowering and sex related genes annotated in the transcriptome of Purple Kush (PK) from (van Bakel et al., 2011). Fragments of the genes can map in different scaffolds. Bp stands for base pair and asterisk (*) stands for lack missing values.

| Flowering related genes | Function description | Genbank accession | SeqName | Transcript name in vanBakel 2011 | Scaffold vanBakel2011 | Transcript (bp) | Start...End |
| --- | --- | --- | --- | --- | --- | --- | --- |
| 14-3-3 16R | Florigen Hd3a interacts with 14-3-3 | GI:351614898 | JP469199.1 | PK02214.1 | scaffold2734 | 1270 | 0...1270 |
| 14-3-3 32kDa endonuclease | Florigen Hd3a interacts with 14-3-3 | GI:351617905 | JP472206.1 | PK06007.1 | scaffold18279 | 1365 | 0...1365 |
| 14-3-3 A | Florigen Hd3a interacts with 14-3-3 | GI:351617070 | JP471371.1 | PK17651.1 | C32009575 | 1217 | 0...460 |
|  |  |  |  |  | scaffold30769 | 1217 | 0...1217 |
| 14-3-3 B | Florigen Hd3a interacts with 14-3-3 | GI:351612187 | JP466488.1 | PK06467.1 | scaffold6434 | 1113 | 0...1112 |
| 14-3-3 D isoform X5 | Florigen Hd3a interacts with 14-3-3 | GI:351613666 | JP467967.1 | PK16733.1 | scaffold18336 | 777 | 0...122 |
|  |  |  |  |  | scaffold31290 | 777 | 0...518 |
|  |  |  |  |  | scaffold61484 | 777 | 515...776 |
| 14-3-3 GF14 iota | Florigen Hd3a interacts with 14-3-3 | GI:351613753 | JP468054.1 | PK02067.1 | scaffold41530 | 1228 | 235...1228 |
|  |  |  |  |  | scaffold82662 | 1228 | 25...545 |
| 14-3-3 GF14 iota | Florigen Hd3a interacts with 14-3-3 | GI:351619282 | JP473583.1 | PK02067.2 | scaffold41530 | 509 | 0...509 |
| 14-3-3 GF14 kappa | Florigen Hd3a interacts with 14-3-3 | GI:351602615 | JP457036.1 | PK13161.2 | scaffold6327 | 830 | 0...830 |
| 14-3-3 GF14 kappa isoform X1 | Florigen Hd3a interacts with 14-3-3 | GI:351615236 | JP469537.1 | PK13161.1 | scaffold6327 | 863 | 0...863 |
| 14-3-3 partial | Florigen Hd3a interacts with 14-3-3 | GI:351607558 | JP461878.1 | PK15983.3 | scaffold30864 | 472 | 0...472 |
| 14-3-3-like protein | Florigen Hd3a interacts with 14-3-3 | GI:351628140 | JP481023.1 | PK06007.2 | scaffold18279 | 524 | 26...523 |
| Actin-related 7 | Seed dormancy & flower abscission | GI:351628659 | JP481542.1 | PK24284.1 | scaffold458 | 1674 | 25...1662 |
|  |  |  |  |  | scaffold17015 | 1674 | 25...1662 |
| Agamous-like MADS-box AGL1 | Candidates for SUPPRESSOR OF OVEREXPRESSION OF CO 1 (SOC1) | GI:351620258 | JP474559.1 | PK01025.1 | scaffold32252 | 851 | 461...851 |
|  |  |  |  |  | scaffold45279 | 851 | 0...465 |
|  |  |  |  |  | scaffold100414 | 851 | 0...383 |
| Agamous-like MADS-box AGL104 | Meristem identity genes_transcription factors | GI:351621137 | JP475438.1 | PK24279.1 | scaffold7284 | 223 | 0...223 |
| Agamous-like MADS-box AGL11 isoform X1 | Meristem identity genes_transcription factors | GI:351624287 | JP478588.1 | PK24532.1 | scaffold24955 | 1154 | 25...1154 |
| Agamous-like MADS-box AGL12 | Meristem identity genes_transcription factors | GI:351622176 | JP476477.1 | PK26896.1 | scaffold14172 | 991 | 0...991 |
| Agamous-like MADS-box AGL15 | Meristem identity genes_transcription factors | GI:351619141 | JP473442.1 | PK08595.1 | scaffold18752 | 705 | 269...661 |
|  |  |  |  |  | scaffold44451 | 705 | 163...302 |
|  |  |  |  |  | scaffold56526 | 705 | 0...168 |
|  |  |  |  |  | scaffold91646 | 705 | 441...705 |
| Agamous-like MADS-box AGL16 | Meristem identity genes_transcription factors | GI:351629150 | JP482033.1 | PK19764.1 | * | * | * |
| Agamous-like MADS-box AGL19 | Meristem identity genes_transcription factors | GI:351620985 | JP475286.1 | PK12001.1 | scaffold87 | 921 | 532...921 |
|  |  |  |  |  | scaffold11630 | 921 | 577...921 |
|  |  |  |  |  | scaffold142350 | 921 | 0...534 |
| Agamous-like MADS-box AGL30 isoform X1 | Meristem identity genes_transcription factors | GI:351621394 | JP475695.1 | PK03147.1 | scaffold9842 | 1733 | 0...1652 |
| Agamous-like MADS-box AGL6 | Meristem identity genes_transcription factors | GI:351625783 | JP480084.1 | PK14825.2 | scaffold5190 | 251 | 24...250 |
| Agamous-like MADS-box AGL9 | Meristem identity genes_transcription factors | GI:351622338 | JP476639.1 | PK19420.1 | scaffold27691 | 1157 | 0...307 |
|  |  |  |  |  | scaffold29034 | 1157 | 305...1157 |
| Agamous-like MADS-box AGL93 | Meristem identity genes_transcription factors | GI:351620903 | JP475204.1 | PK17906.1 | scaffold21952 | 831 | 0...386 |
|  |  |  |  |  | scaffold21952 | 831 | 0...810 |
| Auxin response factor | Auxin signaling pathway | GI:351616372 | JP470673.1 | PK18282.1 | C31901985 | 3259 | 0...218 |
|  |  |  |  |  | scaffold1122 | 3259 | 179...1608 |
|  |  |  |  |  | scaffold22135 | 3259 | 0...3259 |
| Auxin response factor 18 | Auxin signaling pathway | GI:351618517 | JP472818.1 | PK17007.1 | C31998985 | 2755 | 336...1184 |
|  |  |  |  |  | scaffold147078 | 2755 | 0...1496 |
|  |  |  |  |  | scaffold147079 | 2755 | 1145...2755 |
| Auxin response factor 18-like | Auxin signaling pathway | GI:351623409 | JP477710.1 | PK10136.1 | C31900289 | 2180 | 1080...1249 |
|  |  |  |  |  | C31992419 | 2180 | 48...871 |
|  |  |  |  |  | scaffold44146 | 2180 | 888...1084 |
|  |  |  |  |  | scaffold72891 | 2180 | 1033...2180 |
|  |  |  |  |  | scaffold124657 | 2180 | 1527...2180 |
| Auxin response factor 1-like | Auxin signaling pathway | GI:351611621 | JP465922.1 | PK12669.1 | scaffold14169 | 1572 | 3...1572 |
|  |  |  |  |  | scaffold31647 | 1572 | 3...1572 |
| Auxin response factor 2B-like isoform X1 | Auxin signaling pathway | GI:351622980 | JP477281.1 | PK04762.1 | scaffold16869 | 2725 | 0...2724 |
| Auxin response factor 4 isoform X1 | Auxin signaling pathway | GI:351617900 | JP472201.1 | PK07904.1 | scaffold78793 | 2924 | 0...2924 |
|  |  |  |  |  | scaffold88525 | 2924 | 1107...2924 |
| Auxin response factor 5 | Auxin signaling pathway | GI:351620465 | JP474766.1 | PK19328.1 | scaffold21255 | 2904 | 0...2904 |
| Auxin response factor 5 | Auxin signaling pathway | GI:351620161 | JP474462.1 | PK19762.1 | C31900633 | 4137 | 275...799 |
|  |  |  |  |  | C32049703 | 4137 | 3191...3985 |
|  |  |  |  |  | scaffold50702 | 4137 | 0...4070 |
|  |  |  |  |  | scaffold94523 | 4137 | 0...3648 |
|  |  |  |  |  | scaffold138801 | 4137 | 798...2808 |
|  |  |  |  |  | scaffold157023 | 4137 | 4031...4137 |
| Auxin response factor 6 | Auxin signaling pathway | GI:351620418 | JP474719.1 | PK04994.1 | C32006953 | 2267 | 1152...1835 |
|  |  |  |  |  | scaffold35570 | 2267 | 0...2267 |
|  |  |  |  |  | scaffold96684 | 2267 | 0...2267 |
| B3 domain-containing transcription factor VRN1 | Transcriptional repressor of FLC | GI:351618361 | JP472662.1 | PK00037.1 | * | * | * |
| B3 domain-containing transcription factor VRN1 | Transcriptional repressor of FLC | GI:351625424 | JP479725.1 | PK10243.1 | C32014523 | 1907 | 124...674 |
|  |  |  |  |  | C32036921 | 1907 | 845...1560 |
|  |  |  |  |  | scaffold58294 | 1907 | 0...1907 |
|  |  |  |  |  | scaffold58294 | 1907 | 783...940 |
| B3 domain-containing transcription factor VRN1 | Transcriptional repressor of FLC | GI:351628701 | JP481584.1 | PK28451.1 | C31870405 | 1439 | 18...207 |
|  |  |  |  |  | scaffold1994 | 1439 | 1132...1255 |
|  |  |  |  |  | scaffold49208 | 1439 | 368...1438 |
|  |  |  |  |  | scaffold51918 | 1439 | 1085...1255 |
|  |  |  |  |  | scaffold57757 | 1439 | 1085...1255 |
|  |  |  |  |  | scaffold100276 | 1439 | 660...1255 |
| B3 domain-containing transcription factor VRN1 isoform X1 | Transcriptional repressor of FLC | GI:351629415 | JP482298.1 | PK00037.2 | * | * | * |
| B3 domain-containing transcription factor VRN1 isoform X1 | Transcriptional repressor of FLC | GI:351621869 | JP476170.1 | PK09356.1 | scaffold36224 | 706 | 0...706 |
|  |  |  |  |  | scaffold40477 | 706 | 285...706 |
| B3 domain-containing transcription factor VRN1-like | Transcriptional repressor of FLC | GI:351623842 | JP478143.1 | PK05967.1 | scaffold21221 | 243 | 0...243 |
| B3 domain-containing transcription factor VRN1-like | Transcriptional repressor of FLC | GI:351606598 | JP460925.1 | PK09138.1 | scaffold21221 | 558 | 0...558 |
| B3 domain-containing transcription factor VRN1-like | Transcriptional repressor of FLC | GI:351617584 | JP471885.1 | PK10583.1 | C31672876 | 944 | 533...925 |
|  |  |  |  |  | scaffold66943 | 944 | 328...944 |
| B3 domain-containing transcription factor VRN1-like | Transcriptional repressor of FLC | GI:351613639 | JP467940.1 | PK10791.1 | C31892917 | 269 | 10...279 |
|  |  |  |  |  | C32089743 | 1776 | 18...1794 |
|  |  |  |  |  | scaffold113346 | 269 | 3868...4137 |
|  |  |  |  |  | scaffold2205 | 1576 | 21493...23069 |
| B3 domain-containing transcription factor VRN1-like | Transcriptional repressor of FLC | GI:351618817 | JP473118.1 | PK18132.1 | scaffold7951 | 971 | 24...971 |
| B3 domain-containing transcription factor VRN1-like | Transcriptional repressor of FLC | GI:351620946 | JP475247.1 | PK27196.1 | C31817693 | 406 | 147...406 |
|  |  |  |  |  | scaffold79535 | 406 | 0...148 |
|  |  |  |  |  | scaffold119988 | 406 | 0...351 |
| Blue-light photoreceptor PHR2 | Photoreceptors | GI:351621525 | JP475826.1 | PK00736.2 | C31887563 | 1926 | 0...503 |
|  |  |  |  |  | scaffold22601 | 1926 | 294...1926 |
|  |  |  |  |  | scaffold50424 | 1926 | 208...1926 |
| bZIP transcription factor 11-like | A bZIP transcription factor, FD, required for FT to promote flowering | GI:351620593 | JP474894.1 | PK14065.1 | scaffold15368 | 807 | 0...807 |
| bZIP transcription factor 16-like | A bZIP transcription factor, FD, required for FT to promote flowering | GI:351623588 | JP477889.1 | PK23964.1 | C32043703 | 1919 | 874...1214 |
|  |  |  |  |  | scaffold16340 | 1919 | 1211...1919 |
|  |  |  |  |  | scaffold48777 | 1919 | 0...875 |
|  |  |  |  |  | scaffold109857 | 1919 | 1415...1919 |
|  |  |  |  |  | scaffold147427 | 1919 | 238...422 |
| bZIP transcription factor 27-like | A bZIP transcription factor, FD, required for FT to promote flowering | GI:351621867 | JP476168.1 | PK08841.1 | scaffold2448 | 725 | 0...725 |
| bZIP transcription factor 60-like | A bZIP transcription factor, FD, required for FT to promote flowering | GI:351622583 | JP476884.1 | PK05989.1 | scaffold37369 | 958 | 0...958 |
|  |  |  |  |  | scaffold58960 | 958 | 0...958 |
| Cryptochrome 1 family | Photoreceptors | GI:351629217 | JP482100.1 | PK19567.1 | scaffold11691 | 2931 | 0...2931 |
| Cryptochrome chloroplastic mitochondrial | Photoreceptors | GI:351627764 | JP480647.1 | PK06921.1 | scaffold37765 | 2077 | 25...2076 |
| Cryptochrome chloroplastic mitochondrial | Photoreceptors | GI:351601830 | JP456268.1 | PK06921.2 | scaffold37765 | 658 | 0...658 |
| Cryptochrome DNA photolyase class | Photoreceptors | GI:351615213 | JP469514.1 | PK06921.3 | scaffold37765 | 426 | 0...388 |
| Cryptochrome-1 isoform X2 | Photoreceptors | GI:351623616 | JP477917.1 | PK24149.1 | C32046447 | 2402 | 0...479 |
|  |  |  |  |  | scaffold69164 | 2402 | 106...2402 |
|  |  |  |  |  | scaffold89336 | 2402 | 1406...2402 |
|  |  |  |  |  | scaffold119034 | 2402 | 478...702 |
|  |  |  |  |  | scaffold138473 | 2402 | 1684...2078 |
| DELLA RGL1-like | Repressor of the gibberellin (GA) signaling pathway | GI:351618346 | JP472647.1 | PK09859.1 | scaffold31176 | 1167 | 0...1167 |
|  |  |  |  |  | scaffold93902 | 1167 | 0...1167 |
| DELLA RGL1-like | Repressor of the gibberellin (GA) signaling pathway | GI:351620622 | JP474923.1 | PK14882.1 | C32116483 | 1497 | 0...1496 |
| DELLA RGL1-like | Repressor of the gibberellin (GA) signaling pathway | GI:351624825 | JP479126.1 | PK18087.1 | * | * | * |
| DELLA RGL1-like | Repressor of the gibberellin (GA) signaling pathway | GI:351622553 | JP476854.1 | PK23183.1 | scaffold57320 | 402 | 0...402 |
| DELLA RGL1-like | Repressor of the gibberellin (GA) signaling pathway | GI:351604790 | JP459139.1 | PK25517.1 | scaffold57320 | 415 | 13...408 |
|  |  |  |  |  | scaffold68716 | 415 | 13...408 |
| DELLA SLN1-like | ABA-ethylene antagonism, seed dormancy | GI:351618341 | JP472642.1 | PK11483.1 | C32003603 | 1800 | 1373...1800 |
|  |  |  |  |  | C32056853 | 1800 | 0...1216 |
|  |  |  |  |  | scaffold55579 | 1800 | 329...1654 |
| DELLA transcription factor (RGA1-homolog)(putative SpRGI-homolog) | Rapidly degraded in response to GA. Involved in fruit and flower development. | GI:351598083 | JP452616.1 | PK14700.1 | C32094111 | 2382 | 123...2100 |
|  |  |  |  |  | scaffold1196 | 2382 | 2061...2318 |
|  |  |  |  |  | scaffold13394 | 2382 | 10...153 |
| Developmental SEPALLATA 1 | SEPALLATA | GI:351622516 | JP476817.1 | PK10580.1 | scaffold37149 | 717 | 0...717 |
| Developmental SEPALLATA 1 isoform X2 | SEPALLATA | GI:351606046 | JP460383.1 | PK08909.1 | C32020747 | 964 | 433...548 |
|  |  |  |  |  | scaffold7866 | 964 | 0...936 |
|  |  |  |  |  | scaffold10879 | 964 | 717...916 |
|  |  |  |  |  | scaffold71379 | 964 | 12...223 |
|  |  |  |  |  | scaffold97763 | 964 | 221...964 |
| Developmental SEPALLATA 1 isoform X2 | SEPALLATA | GI:351590913 | JP449370.1 | PK08909.2 | C32020747 | 1232 | 620...729 |
|  |  |  |  |  | scaffold7866 | 1232 | 408...1092 |
|  |  |  |  |  | scaffold7866 | 1232 | 0...1231 |
|  |  |  |  |  | scaffold71379 | 1232 | 199...410 |
|  |  |  |  |  | scaffold71379 | 1232 | 408...1144 |
| Developmental ULTRAPETALA | Negative regulator of flower development | GI:351609160 | JP463461.1 | PK17374.1 | C31964578 | 622 | 0...622 |
| DOWNSTREAM OF FLC | Three-gene cluster containing FLC, UFC and DFC, which is coordinately regulated in response to vernalization. Not regulated by FLX. | GI:351618400 | JP472701.1 | PK21503.1 | C32091301 | 1490 | 0...1490 |
| DOWNSTREAM OF FLC-like | Three-gene cluster containing FLC, UFC and DFC, which is coordinately regulated in response to vernalization. Not regulated by FLX. | GI:351623323 | JP477624.1 | PK20061.1 | scaffold56241 | 705 | 0...705 |
|  |  |  |  |  | scaffold65808 | 705 | 0...705 |
| EARLY FLOWERING | Circadian clock, photoperiodism, flowering | GI:351600900 | JP455358.1 | PK21351.2 | scaffold14404 | 1627 | 0...1627 |
| EARLY FLOWERING 4 | Circadian clock, photoperiodism, flowering | GI:351623766 | JP478067.1 | PK14282.2 | C32055425 | 839 | 0...535 |
|  |  |  |  |  | scaffold1822 | 839 | 0...839 |
|  |  |  |  |  | scaffold75548 | 839 | 0...839 |
| ELF4-LIKE 3 | Circadian clock, photoperiodism, flowering | GI:351620039 | JP474340.1 | PK13217.1 | scaffold60665 | 741 | 73...741 |
| ELF4-LIKE 4-like | Circadian clock, photoperiodism, flowering | GI:351615075 | JP469376.1 | PK16816.1 | scaffold25879 | 822 | 0...822 |
|  |  |  |  |  | scaffold25879 | 822 | 0...330 |
| ethylene receptor | Ethylene signaling | GI:351628658 | JP481541.1 | PK14437.1 | C32055281 | 3095 | 2125...3094 |
|  |  |  |  |  | scaffold1168 | 3095 | 44...1166 |
|  |  |  |  |  | scaffold21464 | 3095 | 1127...2369 |
|  |  |  |  |  | scaffold21464 | 3095 | 1161...3094 |
| FLC EXPRESSOR (FLX) | Regulation of FLC | GI:351604611 | JP458961.1 | PK07597.1 | scaffold49139 | 608 | 424...608 |
|  |  |  |  |  | scaffold123126 | 608 | 0...608 |
| FLC EXPRESSOR (FLX) | Regulation of FLC | GI:351616774 | JP471075.1 | PK23868.1 | scaffold123126 | 447 | 0...447 |
| FLC EXPRESSOR isoform X1 (FLX) | Regulation of FLC | GI:351629316 | JP482199.1 | PK07597.2 | C32070477 | 599 | 345...598 |
|  |  |  |  |  | scaffold123126 | 599 | 0...598 |
| Floral homeotic APETALA 2 | Meristem identity genes_transcription factors | GI:351615181 | JP469482.1 | PK17957.1 | scaffold4271 | 369 | 0...369 |
|  |  |  |  |  | scaffold73861 | 369 | 0...369 |
| Floral homeotic APETALA 2 (AP2) | Meristem identity genes_transcription factors | GI:351621780 | JP476081.1 | PK02403.1 | scaffold10879 | 2217 | 0...2217 |
| floral homeotic APETALA 2 isoform X1 | Meristem identity genes_transcription factors | GI:351611084 | JP465385.1 | PK16652.1 | scaffold4271 | 538 | 0...538 |
|  |  |  |  |  | scaffold73861 | 538 | 0...422 |
| Floral homeotic GLOBOSA (GLO) | Meristem identity genes_transcription factors | GI:351621377 | JP475678.1 | PK27392.1 | scaffold3736 | 681 | 0...681 |
|  |  |  |  |  | scaffold103796 | 681 | 0...681 |
| floral homeotic PMADS 2 (flower development) | Meristem identity genes_transcription factors | GI:351620254 | JP474555.1 | PK22420.1 | scaffold77316 | 606 | 257...606 |
|  |  |  |  |  | scaffold92751 | 606 | 0...259 |
| Floricaula leafy (flower and leaf development) | Meristem identity genes_transcription factors | GI:351608783 | JP463087.1 | PK10917.1 | scaffold102797 | 299 | 0...299 |
| FLOWERING LOCUS D (FLD) | Autonomous flowering pathway; inhibiting FLC | GI:351611465 | JP465766.1 | PK11476.2 | scaffold19844 | 872 | 0...872 |
|  |  |  |  |  | scaffold44771 | 872 | 0...872 |
| Flowering locus K homology domain-like | Autonomous flowering pathway; inhibiting FLC | GI:351623152 | JP477453.1 | PK16571.1 | C31919431 | 1106 | 0...479 |
|  |  |  |  |  | scaffold29453 | 1106 | 476...966 |
|  |  |  |  |  | scaffold46899 | 1106 | 757...1102 |
|  |  |  |  |  | scaffold102253 | 1106 | 476...700 |
| Flowering locus k -likey domain | Autonomous flowering pathway; inhibiting FLC | GI:351611055 | JP465356.1 | PK27581.1 | scaffold51716 | 256 | 0...256 |
| Flowering locus T | Regulation of flower development; Florigen-like (PEBP domain; phosphatidylethanolamine binding) | GI:351617403 | JP471704.1 | PK08698.1 | scaffold24080 | 457 | 318...457 |
|  |  |  |  |  | scaffold87765 | 457 | 0...457 |
| Flowering locus T | Regulation of flower development; Florigen-like (PEBP domain; phosphatidylethanolamine binding) | GI:351606973 | JP461297.1 | PK10393.1 | C31892253 | 585 | 290...585 |
|  |  |  |  |  | scaffold5866 | 585 | 0...252 |
| Flowering time control FCA- partial | Autonomous flowering pathway; inhibiting FLC | GI:351622328 | JP476629.1 | PK18808.1 | C31995741 | 1919 | 709...915 |
|  |  |  |  |  | scaffold5756 | 1919 | 1641...1919 |
|  |  |  |  |  | scaffold58436 | 1919 | 0...530 |
|  |  |  |  |  | scaffold143283 | 1919 | 912...1644 |
| Flowering time control FPA | Autonomous flowering pathway; inhibiting FLC | GI:351627999 | JP480882.1 | PK00353.1 | scaffold6692 | 3193 | 0...3184 |
|  |  |  |  |  | scaffold14997 | 3193 | 0...564 |
|  |  |  |  |  | scaffold72038 | 3193 | 562...1796 |
|  |  |  |  |  | scaffold111671 | 3193 | 2558...3184 |
| Flowering time control FPA | Autonomous flowering pathway; inhibiting FLC | GI:351620996 | JP475297.1 | PK00353.2 | scaffold6692 | 438 | 0...438 |
|  |  |  |  |  | scaffold14997 | 438 | 0...438 |
| flowering time control FPA-like | Autonomous flowering pathway; inhibiting FLC | GI:351611268 | JP465569.1 | PK10508.2 | scaffold43284 | 451 | 0...451 |
|  |  |  |  |  | scaffold72756 | 451 | 0...451 |
| Flowering time control FY | Autonomous flowering pathway; inhibiting FLC | GI:351624145 | JP478446.1 | PK04205.1 | scaffold13375 | 2589 | 0...2563 |
| flowering time control partial | Autonomous flowering pathway; inhibiting FLC | GI:351617416 | JP471717.1 | PK12609.1 | scaffold10005 | 2186 | 0...2186 |
| Flowering-promoting factor 1 | Involved in a GA-dependent response in apical meristems during the transition to flowering. Modulates the competence to flowering of apical meristems | GI:351598388 | JP452913.1 | PK26074.1 | scaffold160284 | 491 | 0...490 |
| Flowering-promoting factor 1 (FPF1) | Involved in a GA-dependent response in apical meristems during the transition to flowering. Modulates the competence to flowering of apical meristems | GI:351609258 | JP463559.1 | PK14206.1 | scaffold160572 | 547 | 0...547 |
| Formin 14 isoform X3 | Circadian clock, photoperiodism, flowering | GI:351595682 | JP450247.1 | PK29228.1 | scaffold10275 | 433 | 0...423 |
| Gibberellin 2-beta-dioxygenase 1 | GA balance | GI:351599411 | JP453907.1 | PK28902.1 | scaffold5190 | 1193 | 27...1193 |
| Gibberellin 2-beta-dioxygenase 8 | GA balance | GI:351618701 | JP473002.1 | PK04992.1 | scaffold1471 | 1498 | 792...1498 |
|  |  |  |  |  | scaffold2143 | 1498 | 0...175 |
|  |  |  |  |  | scaffold87163 | 1498 | 789...1176 |
|  |  |  |  |  | scaffold87164 | 1498 | 0...791 |
| Gibberellin 2-beta-dioxygenase 8 | GA balance | GI:351597605 | JP452148.1 | PK18207.1 | scaffold63390 | 1314 | 0...1313 |
|  |  |  |  |  | scaffold140750 | 1314 | 0...320 |
| Gibberellin 2-beta-dioxygenase 8 | GA balance | GI:351597681 | JP452222.1 | PK20950.2 | scaffold21139 | 474 | 0...473 |
| Gibberellin 3-beta-dioxygenase 1 | GA balance | GI:351628803 | JP481686.1 | PK15189.1 | scaffold44826 | 1365 | 716...1365 |
|  |  |  |  |  | scaffold51054 | 1365 | 58...1365 |
|  |  |  |  |  | scaffold51142 | 1365 | 0...1365 |
| Gibberellin 3-beta-dioxygenase 3 | GA balance | GI:351595992 | JP450551.1 | PK00796.1 | scaffold20380 | 1139 | 263...1138 |
|  |  |  |  |  | scaffold43825 | 1139 | 23...538 |
|  |  |  |  |  | scaffold62829 | 1139 | 537...1138 |
| Gibberellin 3-beta-dioxygenase 4 | GA balance | GI:351622209 | JP476510.1 | PK17654.1 | scaffold44816 | 423 | 0...390 |
| Gibberellin receptor GID1B-like | Gibberellin mediated signaling | GI:351619561 | JP473862.1 | PK07006.1 | scaffold27286 | 2392 | 0...2392 |
| Gibberellin receptor GID1C | Gibberellin mediated signaling | GI:351615880 | JP470181.1 | PK14609.1 | C32075887 | 1964 | 369...1812 |
|  |  |  |  |  | scaffold46035 | 1964 | 0...371 |
|  |  |  |  |  | scaffold160637 | 1964 | 1772...1964 |
| Gibberellin receptor GID1C isoform X1 | Gibberellin mediated signaling | GI:351600850 | JP455309.1 | PK14609.2 | C32075887 | 511 | 54...511 |
| Gibberellin regulated | GA balance | GI:351611997 | JP466298.1 | PK04526.1 | scaffold96210 | 402 | 202...402 |
|  |  |  |  |  | scaffold112249 | 402 | 0...402 |
| Gibberellin regulated | GA balance | GI:351610628 | JP464929.1 | PK08429.1 | scaffold67075 | 580 | 0...580 |
| Gibberellin regulated | GA balance | GI:351598982 | JP453487.1 | PK08435.1 | scaffold6298 | 492 | 13...492 |
| Gibberellin regulated | GA balance | GI:351616682 | JP470983.1 | PK09649.1 | scaffold32945 | 405 | 242...405 |
|  |  |  |  |  | scaffold52381 | 405 | 0...405 |
| Gibberellin-2 oxidase | GA balance | GI:351597267 | JP451816.1 | PK05523.1 | scaffold12679 | 1112 | 358...1112 |
|  |  |  |  |  | scaffold93690 | 1112 | 0...363 |
|  |  |  |  |  | scaffold133252 | 1112 | 358...1112 |
| Gibberellin-2 oxidase | GA balance | GI:351604607 | JP458957.1 | PK05523.2 | scaffold93690 | 413 | 0...413 |
| Gibberellin-2 oxidase | GA balance | GI:351604476 | JP458827.1 | PK12475.1 | C32086911 | 1427 | 24...1071 |
|  |  |  |  |  | scaffold66909 | 1427 | 24...1235 |
| Gibberellin-2 oxidase | GA balance | GI:351601075 | JP455528.1 | PK12475.2 | C32086911 | 338 | 0...194 |
|  |  |  |  |  | scaffold66909 | 338 | 0...338 |
| Gibberellin-2 oxidase | GA balance | GI:351596745 | JP451300.1 | PK24354.1 | scaffold4615 | 1232 | 0...1232 |
| Gibberellin-20 oxidase | GA balance | GI:351615475 | JP469776.1 | PK04407.1 | scaffold5190 | 1584 | 0...1584 |
| Gibberellin-20 oxidase | GA balance | GI:351600530 | JP454997.1 | PK04407.2 | scaffold5190 | 999 | 0...999 |
| Gibberellin-20 oxidase | GA balance | GI:351596173 | JP450731.1 | PK18415.1 | scaffold5369 | 964 | 720...964 |
|  |  |  |  |  | scaffold37138 | 964 | 26...964 |
|  |  |  |  |  | scaffold117918 | 964 | 26...401 |
|  |  |  |  |  | scaffold154871 | 964 | 398...722 |
| Gibberellin-regulated 1 | GA balance | GI:351611875 | JP466176.1 | PK11079.1 | scaffold6298 | 358 | 0...358 |
| Gibberellin-regulated 14 isoform X2 | GA balance | GI:351619931 | JP474232.1 | PK16874.1 | C31846133 | 1003 | 532...896 |
|  |  |  |  |  | scaffold28176 | 1003 | 0...534 |
|  |  |  |  |  | scaffold94912 | 1003 | 0...534 |
| Gibberellin-regulated 5 | GA balance | GI:351623566 | JP477867.1 | PK06262.1 | scaffold87095 | 503 | 0...503 |
| Gibberellin-regulated 6 | GA balance | GI:351617498 | JP471799.1 | PK24353.1 | scaffold3335 | 756 | 0...756 |
| Gibberellin-regulated family | GA balance | GI:351619338 | JP473639.1 | PK08957.1 | scaffold44219 | 632 | 0...632 |
|  |  |  |  |  | scaffold59082 | 632 | 0...632 |
| GIGANTEA isoform X1 | Circadian clock, photoperiodism, flowering | GI:351627585 | JP480468.1 | PK12404.1 | C31880379 | 4380 | 3263...3534 |
|  |  |  |  |  | C32002743 | 4380 | 288...527 |
|  |  |  |  |  | C32004153 | 4380 | 3638...4380 |
|  |  |  |  |  | C32096001 | 4380 | 1509...3268 |
|  |  |  |  |  | scaffold37899 | 4380 | 0...1510 |
|  |  |  |  |  | scaffold44163 | 4380 | 524...4380 |
|  |  |  |  |  | scaffold44163 | 4380 | 3693...4028 |
|  |  |  |  |  | scaffold100658 | 4380 | 0...684 |
|  |  |  |  |  | scaffold127736 | 4380 | 3495...3800 |
| GIGANTEA isoform X3 | Circadian clock, photoperiodism, flowering | GI:351606293 | JP460622.1 | PK03725.1 | scaffold141845 | 201 | 70...201 |
| GIGANTEA-like isoform X2 | Circadian clock, photoperiodism, flowering | GI:351609170 | JP463471.1 | PK23071.1 | C31969072 | 289 | 9...289 |
| Glutamate carboxypeptidase 2 | Ethylene signaling | GI:351613638 | JP467939.1 | PK23602.2 | scaffold31758 | 227 | 0...227 |
| HEADING DATE 3A-like | Regulation of flower development; Florigen-like (PEBP domain; phosphatidylethanolamine binding) | GI:351600382 | JP454851.1 | PK10393.2 | C31892253 | 263 | 0...263 |
| HEADING DATE 3A-like | Regulation of flower development; Florigen-like (PEBP domain; phosphatidylethanolamine binding) | GI:351621810 | JP476111.1 | PK16758.1 | scaffold3201 | 588 | 0...588 |
| HEADING DATE 3B-like isoform X1 | Regulation of flower development; Florigen-like (PEBP domain; phosphatidylethanolamine binding) | GI:351627520 | JP480403.1 | PK18215.1 | C31968120 | 2964 | 332...799 |
|  |  |  |  |  | C32051379 | 2964 | 783...1580 |
|  |  |  |  |  | scaffold34385 | 2964 | 783...2950 |
|  |  |  |  |  | scaffold94004 | 2964 | 15...2963 |
| IDA-LIKE 4 (floral abscission) | Seed dormancy & flower abscission | GI:351609528 | JP463829.1 | PK12307.1 | scaffold20940 | 618 | 0...618 |
| Leucine-rich repeat receptor-like serine threonine- kinase BAM1 | Floral organ development | GI:351603744 | JP458100.1 | PK15085.1 | scaffold2394 | 4102 | 21...4102 |
| Leucine-rich repeat receptor-like serine threonine- kinase BAM3 isoform X1 | Floral organ development | GI:351616179 | JP470480.1 | PK05121.1 | C31957490 | 3391 | 2804...3063 |
|  |  |  |  |  | C32070327 | 3391 | 128...1541 |
|  |  |  |  |  | scaffold49868 | 3391 | 26...2807 |
|  |  |  |  |  | scaffold75641 | 3391 | 3195...3365 |
|  |  |  |  |  | scaffold95980 | 3391 | 3193...3365 |
|  |  |  |  |  | scaffold106871 | 3391 | 3024...3220 |
| MADS-box transcription factor | Other MADS box transcription factors | GI:351610898 | JP465199.1 | PK05662.1 | scaffold7284 | 653 | 0...175 |
|  |  |  |  |  | scaffold15938 | 653 | 170...653 |
| MADS-box transcription factor | Other MADS box transcription factors | GI:351611660 | JP465961.1 | PK05685.1 | scaffold6971 | 409 | 162...409 |
|  |  |  |  |  | scaffold53298 | 409 | 0...164 |
| MADS-box transcription factor | Other MADS box transcription factors | GI:351613169 | JP467470.1 | PK07307.1 | scaffold103796 | 200 | 0...200 |
| MADS-box transcription factor | Other MADS box transcription factors | GI:351615913 | JP470214.1 | PK09312.1 | scaffold5190 | 811 | 0...811 |
| MADS-box transcription factor | Other MADS box transcription factors | GI:351600175 | JP454648.1 | PK15717.1 | C31696163 | 1032 | 905...1032 |
|  |  |  |  |  | scaffold73130 | 1032 | 285...905 |
|  |  |  |  |  | scaffold105482 | 1032 | 285...579 |
|  |  |  |  |  | scaffold112970 | 1032 | 0...287 |
| MADS-box transcription factor | Other MADS box transcription factors | GI:351615522 | JP469823.1 | PK17247.1 | scaffold45420 | 418 | 0...418 |
| MADS-box transcription factor | Other MADS box transcription factors | GI:351615119 | JP469420.1 | PK19352.1 | scaffold40749 | 1159 | 0...1159 |
| MADS-box transcription factor | Other MADS box transcription factors | GI:351610585 | JP464886.1 | PK20581.1 | scaffold43523 | 253 | 0...253 |
| MADS-box transcription factor | Other MADS box transcription factors | GI:351611010 | JP465311.1 | PK21701.1 | scaffold3679 | 451 | 71...260 |
|  |  |  |  |  | scaffold3736 | 451 | 0...451 |
|  |  |  |  |  | scaffold103796 | 451 | 0...451 |
| MADS-box transcription factor | Other MADS box transcription factors | GI:351608269 | JP462578.1 | PK21815.1 | scaffold43586 | 491 | 388...491 |
|  |  |  |  |  | scaffold103301 | 491 | 66...211 |
|  |  |  |  |  | scaffold120113 | 491 | 388...491 |
| MADS-box transcription factor | Other MADS box transcription factors | GI:351600123 | JP454599.1 | PK24544.1 | scaffold4633 | 317 | 0...317 |
| MADS-box transcription factor | Other MADS box transcription factors | GI:351598554 | JP453071.1 | PK26778.1 | scaffold58850 | 798 | 203...352 |
|  |  |  |  |  | scaffold66137 | 798 | 0...145 |
|  |  |  |  |  | scaffold160110 | 798 | 203...629 |
| MADS-box transcription factor | Other MADS box transcription factors | GI:351614404 | JP468705.1 | PK27158.1 | scaffold19315 | 444 | 0...197 |
|  |  |  |  |  | scaffold85660 | 444 | 338...444 |
|  |  |  |  |  | scaffold123650 | 444 | 195...379 |
| MADS-box transcription factor | Other MADS box transcription factors | GI:351616463 | JP470764.1 | PK28134.1 | scaffold63978 | 502 | 0...500 |
| MADS-box transcription factor | Other MADS box transcription factors | GI:351598696 | JP453208.1 | PK28167.1 | scaffold7699 | 905 | 0...903 |
| MADS-box transcription factor | Other MADS box transcription factors | GI:351607979 | JP462291.1 | PK29837.1 | scaffold9054 | 417 | 0...417 |
| MADS-box transcription factor 23 isoform X1 | Other MADS box transcription factors | GI:351604874 | JP459223.1 | PK27820.1 | C32109007 | 625 | 0...307 |
|  |  |  |  |  | scaffold4633 | 625 | 303...625 |
| MADS-box transcription factor 23 isoform X2 | Candidates for SUPPRESSOR OF OVEREXPRESSION OF CO 1 (SOC1) | GI:351617524 | JP471825.1 | PK10594.1 | scaffold20590 | 581 | 0...581 |
| MADS-box transcription factor 23 isoform X4 | Other MADS box transcription factors | GI:351613860 | JP468161.1 | PK07093.2 | scaffold11578 | 384 | 3...384 |
| MADS-box transcription factor 23 isoform X5 | Other MADS box transcription factors | GI:351623973 | JP478274.1 | PK07093.1 | scaffold11578 | 1003 | 0...1003 |
| MADS-box transcription factor ANR1 | Other MADS box transcription factors | GI:351605523 | JP459867.1 | PK17591.1 | scaffold20253 | 213 | 0...213 |
| MADS-box transcription factor ANR1 | Other MADS box transcription factors | GI:351596005 | JP450564.1 | PK25696.1 | scaffold76316 | 370 | 0...369 |
|  |  |  |  |  | scaffold89097 | 370 | 0...369 |
|  |  |  |  |  | scaffold153975 | 370 | 0...369 |
| MADS-box transcription factor; FLC-like | FLOWERING LOCUS C-like | GI:351612809 | JP467110.1 | PK28790.1 | scaffold5000 | 763 | 519...741 |
|  |  |  |  |  | scaffold28498 | 763 | 307...522 |
|  |  |  |  |  | scaffold75283 | 763 | 519...763 |
| MIKC mads-box transcription isoform 1 | Other MADS box transcription factors | GI:351619010 | JP473311.1 | PK11571.1 | scaffold40699 | 794 | 482...734 |
|  |  |  |  |  | scaffold81619 | 794 | 292...765 |
|  |  |  |  |  | scaffold154317 | 794 | 0...212 |
| Phosphatidylethanolamine-binding PEBP | Regulation of flower development; Florigen-like (PEBP domain; phosphatidylethanolamine binding) | GI:351605075 | JP459422.1 | PK05153.1 | scaffold12731 | 343 | 0...343 |
|  |  |  |  |  | scaffold80541 | 343 | 0...343 |
| Phosphatidylethanolamine-binding PEBP | Regulation of flower development; Florigen-like (PEBP domain; phosphatidylethanolamine binding) | GI:351614537 | JP468838.1 | PK13716.1 | scaffold4564 | 958 | 0...958 |
| Phosphatidylethanolamine-binding PEBP | Regulation of flower development; Florigen-like (PEBP domain; phosphatidylethanolamine binding) | GI:351611885 | JP466186.1 | PK15097.1 | scaffold62047 | 926 | 0...926 |
|  |  |  |  |  | scaffold62047 | 926 | 681...926 |
| PHOTOPERIOD-INDEPENDENT EARLY FLOWERING | Autonomous flowering pathway; inhibiting FLC | GI:351606496 | JP460824.1 | PK04588.2 | scaffold29997 | 571 | 246...571 |
| PHOTOPERIOD-INDEPENDENT EARLY FLOWERING | Autonomous flowering pathway; inhibiting FLC | GI:351603969 | JP458324.1 | PK13307.1 | scaffold29997 | 2721 | 2593...2717 |
|  |  |  |  |  | scaffold29997 | 2721 | 0...2718 |
| PHOTOPERIOD-INDEPENDENT EARLY FLOWERING 1 isoform X1 (PIE1) | Autonomous flowering pathway; inhibiting FLC | GI:351622429 | JP476730.1 | PK04588.1 | C31841689 | 1338 | 0...129 |
|  |  |  |  |  | C32076069 | 1338 | 211...760 |
|  |  |  |  |  | scaffold29997 | 1338 | 759...1338 |
| phytochrome A | Photoreceptors | GI:351624418 | JP478719.1 | PK25677.1 | scaffold42686 | 4327 | 0...4321 |
| Phytochrome A B C D E | Photoreceptors | GI:351600623 | JP455087.1 | PK18424.2 | scaffold33564 | 4321 | 0...4321 |
| Phytochrome A B C D E | Photoreceptors | GI:351605782 | JP460124.1 | PK18424.4 | scaffold25104 | 2728 | 51...591 |
|  |  |  |  |  | scaffold33564 | 2728 | 561...2728 |
|  |  |  |  |  | scaffold69789 | 2728 | 51...591 |
| Phytochrome B | Photoreceptors | GI:351624553 | JP478854.1 | PK13238.1 | C31917831 | 4421 | 3308...3783 |
|  |  |  |  |  | C31975762 | 4421 | 2544...3311 |
|  |  |  |  |  | C32037849 | 4421 | 1468...2502 |
|  |  |  |  |  | scaffold98428 | 4421 | 0...1507 |
|  |  |  |  |  | scaffold137277 | 4421 | 3744...4421 |
| Phytochrome E | Photoreceptors | GI:351628146 | JP481029.1 | PK18424.1 | scaffold17828 | 4048 | 24...358 |
|  |  |  |  |  | scaffold33564 | 4048 | 326...4048 |
|  |  |  |  |  | scaffold73428 | 4048 | 24...324 |
| PREDICTED: cullin-4-like (CUL4-like) | Circadian clock, photoperiodism, flowering | GI:351629312 | JP482195.1 | PK28242.1 | C31961490 | 3031 | 1329...1666 |
|  |  |  |  |  | C32019757 | 3031 | 1886...2201 |
|  |  |  |  |  | C32066867 | 3031 | 2198...3031 |
|  |  |  |  |  | scaffold4509 | 3031 | 562...2339 |
|  |  |  |  |  | scaffold79490 | 3031 | 23...1332 |
|  |  |  |  |  | scaffold134539 | 3031 | 23...565 |
|  |  |  |  |  | scaffold143724 | 3031 | 621...1027 |
| Probable lysine-specific demethylase ELF6 | Circadian clock, photoperiodism, flowering | GI:351619606 | JP473907.1 | PK12852.1 | C32110109 | 5322 | 168...2233 |
|  |  |  |  |  | scaffold35877 | 5322 | 0...5321 |
|  |  |  |  |  | scaffold133735 | 5322 | 0...207 |
| Probable UDP-N-acetylglucosamine--peptide N-acetylglucosaminyltransferase SPINDLY | GA balance | GI:351628243 | JP481126.1 | PK20658.1 | scaffold9737 | 3657 | 26...3657 |
| Protein RETICULATA-related (RER) | Photoperiodic growth (Arabidopsis) | GI:351608416 | JP462723.1 | PK16660.1 | scaffold113131 | 1220 | 0...722 |
|  |  |  |  |  | scaffold126147 | 1220 | 719...1220 |
| Protein RETICULATA-related (RER) | Photoperiodic growth (Arabidopsis) | GI:351613103 | JP467404.1 | PK24402.1 | scaffold113131 | 353 | 0...353 |
| Squamosa promoter-binding 1 (SPL1) | SQUAMOSA Promoter-Binding Protein-Like (SPL) genes | GI:351619384 | JP473685.1 | PK06791.1 | C31935432 | 3794 | 2487...2947 |
|  |  |  |  |  | C31955822 | 3794 | 2342...2489 |
|  |  |  |  |  | scaffold42243 | 3794 | 0...2291 |
|  |  |  |  |  | scaffold57634 | 3794 | 2908...3794 |
|  |  |  |  |  | scaffold88031 | 3794 | 0...1320 |
|  |  |  |  |  | scaffold88830 | 3794 | 2908...3695 |
| Squamosa promoter-binding 1 (SPL1) | SQUAMOSA Promoter-Binding Protein-Like (SPL) genes | GI:351602040 | JP456475.1 | PK11310.3 | C32087959 | 624 | 94...624 |
|  |  |  |  |  | scaffold91789 | 624 | 15...624 |
| Squamosa promoter-binding 1 (SPL1) | SQUAMOSA Promoter-Binding Protein-Like (SPL) genes | GI:351628111 | JP465956.1 | PK21111.1 | scaffold123704 | 403 | 44...403 |
|  |  |  |  |  | scaffold123705 | 403 | 44...403 |
| Squamosa promoter-binding 12 (SPL12) | SQUAMOSA Promoter-Binding Protein-Like (SPL) genes | GI:351597361 | JP451909.1 | PK11310.1 | C32087959 | 3167 | 231...1536 |
|  |  |  |  |  | scaffold36354 | 3167 | 0...163 |
|  |  |  |  |  | scaffold61615 | 3167 | 1533...2420 |
|  |  |  |  |  | scaffold91789 | 3167 | 0...763 |
|  |  |  |  |  | scaffold97327 | 3167 | 2416...3164 |
|  |  |  |  |  | scaffold97327 | 3167 | 1533...3164 |
| Squamosa promoter-binding 12 (SPL12) | SQUAMOSA Promoter-Binding Protein-Like (SPL) genes | GI:351598906 | JP453413.1 | PK11310.2 | C32087959 | 959 | 0...928 |
| Squamosa promoter-binding 12 (SPL12) | SQUAMOSA Promoter-Binding Protein-Like (SPL) genes | GI:351615438 | JP469739.1 | PK11310.4 | scaffold61615 | 330 | 24...330 |
|  |  |  |  |  | scaffold97327 | 330 | 24...330 |
| Squamosa promoter-binding 13A (SPL13A) | SQUAMOSA Promoter-Binding Protein-Like (SPL) genes | GI:351608797 | JP463101.1 | PK02140.1 | C32047543 | 821 | 158...689 |
|  |  |  |  |  | scaffold77782 | 821 | 0...821 |
|  |  |  |  |  | scaffold158031 | 821 | 158...821 |
| Squamosa promoter-binding 13A (SPL13A) | SQUAMOSA Promoter-Binding Protein-Like (SPL) genes | GI:351619486 | JP473787.1 | PK27138.1 | scaffold77782 | 927 | 0...927 |
|  |  |  |  |  | scaffold77782 | 927 | 84...245 |
|  |  |  |  |  | scaffold121411 | 927 | 59...927 |
|  |  |  |  |  | scaffold158031 | 927 | 0...927 |
|  |  |  |  |  | scaffold158031 | 927 | 84...245 |
| Squamosa promoter-binding 14 (SPL14) | SQUAMOSA Promoter-Binding Protein-Like (SPL) genes | GI:351602244 | JP456673.1 | PK18312.2 | scaffold72139 | 1146 | 0...1145 |
|  |  |  |  |  | scaffold92998 | 1146 | 754...1145 |
| Squamosa promoter-binding 14 (SPL14) | SQUAMOSA Promoter-Binding Protein-Like (SPL) genes | GI:351597831 | JP452369.1 | PK25661.1 | scaffold13407 | 4432 | 1...4432 |
| Squamosa promoter-binding 16 (SPL16) | SQUAMOSA Promoter-Binding Protein-Like (SPL) genes | GI:351620574 | JP474875.1 | PK14243.1 | scaffold900 | 769 | 0...769 |
| Squamosa promoter-binding 16 (SPL16) | SQUAMOSA Promoter-Binding Protein-Like (SPL) genes | GI:351623164 | JP477465.1 | PK19500.1 | scaffold28707 | 1054 | 0...1033 |
| Squamosa promoter-binding 1-like (SPL1-like) | SQUAMOSA Promoter-Binding Protein-Like (SPL) genes | GI:351623229 | JP477530.1 | PK22320.1 | scaffold4343 | 997 | 0...997 |
| Squamosa promoter-binding 3 (SPL3) | SQUAMOSA Promoter-Binding Protein-Like (SPL) genes | GI:351616382 | JP470683.1 | PK16953.1 | C32084737 | 2840 | 140...1473 |
|  |  |  |  |  | scaffold35463 | 2840 | 0...2840 |
|  |  |  |  |  | scaffold117676 | 2840 | 1469...1916 |
| Squamosa promoter-binding 6 (SPL6) | SQUAMOSA Promoter-Binding Protein-Like (SPL) genes | GI:351598202 | JP452731.1 | PK03565.1 | C31882761 | 1916 | 0...245 |
|  |  |  |  |  | scaffold92933 | 1916 | 244...1916 |
| Squamosa promoter-binding 6 (SPL6) | SQUAMOSA Promoter-Binding Protein-Like (SPL) genes | GI:351614511 | JP468812.1 | PK03565.2 | scaffold92933 | 917 | 0...917 |
| Squamosa promoter-binding 6 (SPL6) | SQUAMOSA Promoter-Binding Protein-Like (SPL) genes | GI:351621002 | JP475303.1 | PK17790.1 | scaffold5882 | 2241 | 0...2241 |
| Squamosa promoter-binding 7 (SPL7) | SQUAMOSA Promoter-Binding Protein-Like (SPL) genes | GI:351605601 | JP459945.1 | PK29723.1 | scaffold33927 | 3346 | 293...2362 |
|  |  |  |  |  | scaffold40883 | 3346 | 2591...3346 |
|  |  |  |  |  | scaffold48023 | 3346 | 1246...1621 |
|  |  |  |  |  | scaffold79549 | 3346 | 22...3346 |
| Squamosa promoter-binding 8 (SPL8) | SQUAMOSA Promoter-Binding Protein-Like (SPL) genes | GI:351612317 | JP466618.1 | PK25779.1 | scaffold122019 | 901 | 0...901 |
| Squamosa promoter-binding 8 (SPL8) | SQUAMOSA Promoter-Binding Protein-Like (SPL) genes | GI:351623221 | JP477522.1 | PK26480.1 | scaffold122019 | 710 | 0...710 |
| SUPPRESSOR OF PHYA-105 1 (SPA1) | Circadian clock, photoperiodism, flowering | GI:351628512 | JP481395.1 | PK13755.1 | scaffold1953 | 3385 | 64...3385 |
|  |  |  |  |  | scaffold55071 | 3385 | 28...159 |
|  |  |  |  |  | scaffold89748 | 3385 | 309...3385 |
| SUPPRESSOR OF PHYA-105 1 (SPA1) | Circadian clock, photoperiodism, flowering | GI:351601725 | JP456166.1 | PK13755.2 | scaffold1953 | 774 | 0...774 |
| TIME FOR COFFEE | Circadian clock, photoperiodism, flowering | GI:351599695 | JP454183.1 | PK04129.1 | C31939040 | 1286 | 1006...1286 |
|  |  |  |  |  | scaffold14899 | 1286 | 685...1286 |
|  |  |  |  |  | scaffold15130 | 1286 | 0...1008 |
| TIME FOR COFFEE | Circadian clock, photoperiodism, flowering | GI:351599306 | JP453803.1 | PK11261.3 | C31939040 | 1991 | 0...251 |
|  |  |  |  |  | C32012205 | 1991 | 1693...1951 |
|  |  |  |  |  | scaffold14899 | 1991 | 0...1721 |
|  |  |  |  |  | scaffold113332 | 1991 | 1813...1991 |
| TIME FOR COFFEE (TIC) | Circadian clock, photoperiodism, flowering | GI:351614086 | JP468387.1 | PK11261.6 | C32058059 | 235 | 0...235 |
|  |  |  |  |  | scaffold14899 | 235 | 0...235 |
| TIME FOR COFFEE isoform X2 | Circadian clock, photoperiodism, flowering | GI:351603388 | JP457746.1 | PK11261.1 | C32058059 | 4226 | 2967...4211 |
|  |  |  |  |  | scaffold14899 | 4226 | 0...4226 |
| TIME FOR COFFEE isoform X3 | Circadian clock, photoperiodism, flowering | GI:351599567 | JP454057.1 | PK04129.2 | C31939040 | 972 | 694...972 |
|  |  |  |  |  | scaffold14899 | 972 | 342...972 |
|  |  |  |  |  | scaffold15130 | 972 | 0...695 |
| Timeless homolog | Circadian clock, photoperiodism, flowering | GI:351598875 | JP453383.1 | PK26798.1 | C31951316 | 4072 | 1420...1588 |
|  |  |  |  |  | C32010955 | 4072 | 432...956 |
|  |  |  |  |  | scaffold17113 | 4072 | 0...1421 |
|  |  |  |  |  | scaffold65509 | 4072 | 0...120 |
|  |  |  |  |  | scaffold73565 | 4072 | 1586...4072 |
|  |  |  |  |  | scaffold104772 | 4072 | 2947...4072 |
|  |  |  |  |  | scaffold125941 | 4072 | 917...1421 |
| Transcriptional regulator | Circadian clock, photoperiodism, flowering | GI:351612262 | JP466563.1 | PK06680.1 | scaffold40086 | 276 | 0...189 |
| ULTRAPETALA 1-like (ULT1) | Negative regulator of flower development | GI:351623359 | JP477660.1 | PK16281.1 | scaffold22960 | 873 | 92...873 |
|  |  |  |  |  | scaffold56652 | 873 | 419...873 |
| Ultraviolet-B receptor UVR8 | Photoreceptors | GI:351591200 | JP449654.1 | PK07299.1 | scaffold46774 | 1835 | 26...1835 |
| Ultraviolet-B receptor UVR8 | Photoreceptors | GI:351614638 | JP468939.1 | PK07299.2 | scaffold46774 | 286 | 42...261 |
| UNUSUAL FLORAL ORGANS-like (UFO-like) | Meristem identity genes_transcription factors | GI:351612959 | JP467260.1 | PK29338.1 | scaffold98732 | 310 | 0...310 |
|  |  |  |  |  | scaffold152035 | 310 | 59...310 |
| UPSTREAM OF FLC | Three-gene cluster containing FLC, UFC and DFC, which is coordinately regulated in response to vernalization. Not regulated by FLX. | GI:351621996 | JP476297.1 | PK01303.1 | C31875855 | 1944 | 467...911 |
|  |  |  |  |  | scaffold8607 | 1944 | 178...1944 |
|  |  |  |  |  | scaffold51718 | 1944 | 23...200 |
|  |  |  |  |  | scaffold74589 | 1944 | 178...495 |
|  |  |  |  |  | scaffold121764 | 1944 | 23...200 |
| UPSTREAM OF FLC | Three-gene cluster containing FLC, UFC and DFC, which is coordinately regulated in response to vernalization. Not regulated by FLX. | GI:351611026 | JP465327.1 | PK01303.3 | C31875855 | 759 | 400...759 |
|  |  |  |  |  | scaffold8607 | 759 | 0...759 |
|  |  |  |  |  | scaffold74589 | 759 | 0...428 |
| UPSTREAM OF FLC | Three-gene cluster containing FLC, UFC and DFC, which is coordinately regulated in response to vernalization. Not regulated by FLX. | GI:351591393 | JP449840.1 | PK23620.1 | scaffold2296 | 1746 | 26...1746 |
|  |  |  |  |  | scaffold133297 | 1746 | 26...1746 |
|  |  |  |  |  | scaffold133297 | 1746 | 98...348 |
| UPSTREAM OF FLC isoform X1 | Three-gene cluster containing FLC, UFC and DFC, which is coordinately regulated in response to vernalization. Not regulated by FLX. | GI:351628106 | JP480989.1 | PK04420.1 | C31917243 | 2041 | 1520...2041 |
|  |  |  |  |  | scaffold17096 | 2041 | 13...1580 |
|  |  |  |  |  | scaffold109476 | 2041 | 13...1782 |
| UPSTREAM OF FLC-like | Three-gene cluster containing FLC, UFC and DFC, which is coordinately regulated in response to vernalization. Not regulated by FLX. | GI:351606503 | JP460831.1 | PK20929.1 | C32027537 | 952 | 0...788 |
|  |  |  |  |  | scaffold16805 | 952 | 0...952 |
|  |  |  |  |  | scaffold41301 | 952 | 610...913 |
| XAP5 CIRCADIAN TIMEKEEPER isoform X2 | Circadian clock, photoperiodism, flowering | GI:351628049 | JP480932.1 | PK10982.1 | scaffold1342 | 1305 | 26...1305 |
| Zinc finger BED domain-containing RICESLEEPER 1-like | Transposase-like protein that is essential for plant growth and development. | GI:351617522 | JP471823.1 | PK11791.1 | scaffold54346 | 735 | 0...735 |
|  |  |  |  |  | scaffold58161 | 735 | 0...735 |
| Zinc finger BED domain-containing RICESLEEPER 1-like | Transposase-like protein that is essential for plant growth and development. | GI:351624405 | JP478706.1 | PK12364.1 | scaffold84456 | 2612 | 24...2612 |
| Zinc finger BED domain-containing RICESLEEPER 1-like | Transposase-like protein that is essential for plant growth and development. | GI:351629060 | JP481943.1 | PK12364.2 | scaffold84456 | 997 | 29...997 |
| Zinc finger BED domain-containing RICESLEEPER 1-like isoform X1 | Transposase-like protein that is essential for plant growth and development. | GI:351629104 | JP481987.1 | PK12133.1 | C32000045 | 1871 | 966...1818 |
|  |  |  |  |  | scaffold86432 | 1871 | 30...139 |
|  |  |  |  |  | scaffold109992 | 1871 | 137...1005 |
| Zinc finger BED domain-containing RICESLEEPER 2 | Transposase-like protein that is essential for plant growth and development. | GI:351625789 | JP480090.1 | PK06118.1 | scaffold15512 | 259 | 0...259 |
| Zinc finger BED domain-containing RICESLEEPER 2-like | Transposase-like protein that is essential for plant growth and development. | GI:351604453 | JP458804.1 | PK01491.1 | scaffold16141 | 2503 | 2227...2381 |
|  |  |  |  |  | scaffold16641 | 2503 | 286...1932 |
|  |  |  |  |  | scaffold40309 | 2503 | 0...219 |
| Zinc finger BED domain-containing RICESLEEPER 2-like | Transposase-like protein that is essential for plant growth and development. | GI:351605013 | JP459361.1 | PK05670.1 | scaffold1221 | 333 | 0...134 |
|  |  |  |  |  | scaffold15512 | 333 | 7...333 |
|  |  |  |  |  | scaffold31703 | 333 | 0...246 |
|  |  |  |  |  | scaffold64764 | 333 | 7...333 |
| Zinc finger BED domain-containing RICESLEEPER 2-like | Transposase-like protein that is essential for plant growth and development. | GI:351624770 | JP479071.1 | PK06251.1 | scaffold1557 | 223 | 0...223 |
|  |  |  |  |  | scaffold4808 | 223 | 57...223 |
|  |  |  |  |  | scaffold5264 | 223 | 0...223 |
|  |  |  |  |  | scaffold57858 | 223 | 0...223 |
| Zinc finger BED domain-containing RICESLEEPER 2-like | Transposase-like protein that is essential for plant growth and development. | GI:351607345 | JP461667.1 | PK07698.1 | * | * | * |
| Zinc finger BED domain-containing RICESLEEPER 2-like | Transposase-like protein that is essential for plant growth and development. | GI:351590822 | JP449280.1 | PK10457.1 | scaffold10189 | 1944 | 0...1944 |
| Zinc finger BED domain-containing RICESLEEPER 2-like | Transposase-like protein that is essential for plant growth and development. | GI:351600842 | JP455301.1 | PK11902.1 | scaffold2026 | 2675 | 489...2675 |
|  |  |  |  |  | scaffold5754 | 2675 | 0...1942 |
|  |  |  |  |  | scaffold9179 | 2675 | 125...2675 |
|  |  |  |  |  | scaffold34735 | 2675 | 2395...2624 |
|  |  |  |  |  | scaffold35830 | 2675 | 52...297 |
|  |  |  |  |  | scaffold35830 | 2675 | 0...956 |
|  |  |  |  |  | scaffold66522 | 2675 | 52...2063 |
|  |  |  |  |  | scaffold82270 | 2675 | 0...542 |
|  |  |  |  |  | scaffold85253 | 2675 | 190...297 |
| Zinc finger BED domain-containing RICESLEEPER 2-like | Transposase-like protein that is essential for plant growth and development. | GI:351604658 | JP459008.1 | PK11902.2 | scaffold2026 | 1782 | 554...924 |
|  |  |  |  |  | scaffold5754 | 1782 | 0...422 |
|  |  |  |  |  | scaffold9179 | 1782 | 99...1782 |
|  |  |  |  |  | scaffold35830 | 1782 | 26...362 |
|  |  |  |  |  | scaffold35830 | 1782 | 0...1021 |
|  |  |  |  |  | scaffold66522 | 1782 | 26...1782 |
|  |  |  |  |  | scaffold82270 | 1782 | 0...607 |
|  |  |  |  |  | scaffold85253 | 1782 | 164...362 |
| Zinc finger BED domain-containing RICESLEEPER 2-like | Transposase-like protein that is essential for plant growth and development. | GI:351625546 | JP479847.1 | PK12026.1 | scaffold9487 | 2508 | 239...2010 |
|  |  |  |  |  | scaffold13327 | 2508 | 239...842 |
|  |  |  |  |  | scaffold17579 | 2508 | 2298...2507 |
|  |  |  |  |  | scaffold18522 | 2508 | 1146...1379 |
|  |  |  |  |  | scaffold22816 | 2508 | 447...2283 |
|  |  |  |  |  | scaffold62185 | 2508 | 1553...1729 |
|  |  |  |  |  | scaffold93354 | 2508 | 1536...1818 |
| Zinc finger BED domain-containing RICESLEEPER 2-like | Transposase-like protein that is essential for plant growth and development. | GI:351602481 | JP456904.1 | PK12682.1 | scaffold481 | 261 | 0...261 |
|  |  |  |  |  | scaffold2082 | 261 | 1...254 |
|  |  |  |  |  | scaffold23228 | 261 | 0...261 |
|  |  |  |  |  | scaffold29022 | 261 | 0...164 |
|  |  |  |  |  | scaffold30382 | 261 | 52...261 |
|  |  |  |  |  | scaffold40620 | 261 | 0...254 |
|  |  |  |  |  | scaffold46569 | 261 | 0...261 |
| Zinc finger BED domain-containing RICESLEEPER 2-like | Transposase-like protein that is essential for plant growth and development. | GI:351617806 | JP472107.1 | PK13280.10 | scaffold13977 | 2450 | 101...1865 |
|  |  |  |  |  | scaffold44696 | 2450 | 101...1860 |
|  |  |  |  |  | scaffold57397 | 2450 | 0...103 |
|  |  |  |  |  | scaffold94980 | 2450 | 743...911 |
| Zinc finger BED domain-containing RICESLEEPER 2-like | Transposase-like protein that is essential for plant growth and development. | GI:351612520 | JP466821.1 | PK13280.12 | scaffold13977 | 1283 | 365...1051 |
|  |  |  |  |  | scaffold44696 | 1283 | 728...1046 |
| Zinc finger BED domain-containing RICESLEEPER 2-like | Transposase-like protein that is essential for plant growth and development. | GI:351621907 | JP476208.1 | PK13949.1 | C32006149 | 2750 | 221...879 |
|  |  |  |  |  | C32036471 | 2750 | 952...2001 |
|  |  |  |  |  | scaffold4156 | 2750 | 2471...2609 |
|  |  |  |  |  | scaffold25486 | 2750 | 2456...2626 |
|  |  |  |  |  | scaffold41216 | 2750 | 2476...2609 |
|  |  |  |  |  | scaffold60538 | 2750 | 1962...2604 |
|  |  |  |  |  | scaffold60539 | 2750 | 1962...2473 |
|  |  |  |  |  | scaffold140373 | 2750 | 25...221 |
| Zinc finger BED domain-containing RICESLEEPER 2-like | Transposase-like protein that is essential for plant growth and development. | GI:351607292 | JP461614.1 | PK15096.1 | scaffold1979 | 212 | 0...212 |
|  |  |  |  |  | scaffold6706 | 212 | 0...212 |
|  |  |  |  |  | scaffold7939 | 212 | 0...169 |
|  |  |  |  |  | scaffold18588 | 212 | 0...212 |
|  |  |  |  |  | scaffold64764 | 212 | 95...211 |
|  |  |  |  |  | scaffold83967 | 212 | 0...212 |
|  |  |  |  |  | scaffold106318 | 212 | 76...212 |
| Zinc finger BED domain-containing RICESLEEPER 2-like | Transposase-like protein that is essential for plant growth and development. | GI:351604841 | JP459190.1 | PK19596.1 | scaffold25978 | 255 | 0...153 |
|  |  |  |  |  | scaffold42928 | 255 | 0...255 |
| Zinc finger BED domain-containing RICESLEEPER 2-like | Transposase-like protein that is essential for plant growth and development. | GI:351601677 | JP456118.1 | PK20445.1 | scaffold7737 | 2759 | 0...2432 |
|  |  |  |  |  | scaffold89213 | 2759 | 2551...2718 |
| Zinc finger BED domain-containing RICESLEEPER 2-like | Transposase-like protein that is essential for plant growth and development. | GI:351598707 | JP453218.1 | PK20445.2 | scaffold7737 | 233 | 3...233 |
| Zinc finger BED domain-containing RICESLEEPER 2-like | Transposase-like protein that is essential for plant growth and development. | GI:351596705 | JP451260.1 | PK21917.1 | scaffold20795 | 998 | 875...998 |
|  |  |  |  |  | scaffold104939 | 998 | 0...998 |
| Zinc finger BED domain-containing RICESLEEPER 2-like | Transposase-like protein that is essential for plant growth and development. | GI:351591415 | JP449862.1 | PK21951.1 | * | * | * |
| Zinc finger BED domain-containing RICESLEEPER 2-like | Transposase-like protein that is essential for plant growth and development. | GI:351618389 | JP472690.1 | PK22698.1 | scaffold7737 | 782 | 0...782 |
| Zinc finger BED domain-containing RICESLEEPER 2-like | Transposase-like protein that is essential for plant growth and development. | GI:351600219 | JP454690.1 | PK25268.1 | scaffold47646 | 447 | 0...447 |
| Zinc finger BED domain-containing RICESLEEPER 2-like | Transposase-like protein that is essential for plant growth and development. | GI:351610670 | JP464971.1 | PK25549.1 | scaffold59729 | 491 | 45...491 |
|  |  |  |  |  | scaffold114541 | 491 | 45...491 |
| Zinc finger BED domain-containing RICESLEEPER 2-like | Transposase-like protein that is essential for plant growth and development. | GI:351606110 | JP460444.1 | PK25767.1 | scaffold1830 | 2860 | 2310...2818 |
|  |  |  |  |  | scaffold2231 | 2860 | 0...168 |
|  |  |  |  |  | scaffold3005 | 2860 | 2496...2823 |
|  |  |  |  |  | scaffold8672 | 2860 | 2672...2823 |
|  |  |  |  |  | scaffold16740 | 2860 | 0...2823 |
|  |  |  |  |  | scaffold20158 | 2860 | 1977...2827 |
|  |  |  |  |  | scaffold23515 | 2860 | 0...2140 |
|  |  |  |  |  | scaffold30553 | 2860 | 2302...2503 |
|  |  |  |  |  | scaffold30969 | 2860 | 0...2823 |
|  |  |  |  |  | scaffold34758 | 2860 | 2616...2823 |
|  |  |  |  |  | scaffold39599 | 2860 | 2562...2827 |
|  |  |  |  |  | scaffold41868 | 2860 | 0...618 |
|  |  |  |  |  | scaffold78776 | 2860 | 2227...2823 |
|  |  |  |  |  | scaffold87322 | 2860 | 1532...2823 |
|  |  |  |  |  | scaffold97069 | 2860 | 2563...2823 |
|  |  |  |  |  | scaffold112221 | 2860 | 1742...2169 |
|  |  |  |  |  | scaffold121204 | 2860 | 1528...1937 |
| Zinc finger BED domain-containing RICESLEEPER 2-like | Transposase-like protein that is essential for plant growth and development. | GI:351604878 | JP459227.1 | PK26091.1 | scaffold8197 | 283 | 0...283 |
| Zinc finger BED domain-containing RICESLEEPER 2-like | Transposase-like protein that is essential for plant growth and development. | GI:351605163 | JP459510.1 | PK26772.1 | scaffold47646 | 241 | 0...241 |
| Zinc finger BED domain-containing RICESLEEPER 2-like | Transposase-like protein that is essential for plant growth and development. | GI:351606593 | JP460920.1 | PK28693.1 | scaffold6706 | 364 | 0...364 |
| Zinc finger BED domain-containing RICESLEEPER 2-like | Transposase-like protein that is essential for plant growth and development. | GI:351604904 | JP459252.1 | PK29123.1 | scaffold35609 | 344 | 1...344 |
| Zinc finger BED domain-containing RICESLEEPER 2-like isoform X2 | Transposase-like protein that is essential for plant growth and development. | GI:351628041 | JP480924.1 | PK11902.3 | * | * | * |
| Zinc finger CONSTANS-LIKE 13 | B-box zinc finger CONSTANS (Arabidopsis); Heading date1 (rice); GmCOL10 (soybean) | GI:351618526 | JP472827.1 | PK04274.1 | scaffold14611 | 1352 | 0...1352 |
|  |  |  |  |  | scaffold35451 | 1352 | 0...1352 |
| Zinc finger CONSTANS-LIKE 14-like | B-box zinc finger CONSTANS (Arabidopsis); Heading date1 (rice); GmCOL10 (soybean) | GI:351591634 | JP450080.1 | PK11450.1 | scaffold14020 | 1867 | 9...1010 |
|  |  |  |  |  | scaffold100392 | 1867 | 9...1867 |
| Zinc finger CONSTANS-LIKE 15 | B-box zinc finger CONSTANS (Arabidopsis); Heading date1 (rice); GmCOL10 (soybean) | GI:351624852 | JP479153.1 | PK17744.1 | scaffold82914 | 2079 | 8...2079 |
|  |  |  |  |  | scaffold84207 | 2079 | 1660...2079 |
| Zinc finger CONSTANS-LIKE 16 | B-box zinc finger CONSTANS (Arabidopsis); Heading date1 (rice); GmCOL10 (soybean) | GI:351618175 | JP472476.1 | PK18183.1 | scaffold12036 | 1750 | 0...1750 |
| Zinc finger CONSTANS-LIKE 2 | B-box zinc finger CONSTANS (Arabidopsis); Heading date1 (rice); GmCOL10 (soybean) | GI:351619363 | JP473664.1 | PK23361.1 | scaffold13708 | 1141 | 0...1141 |
| Zinc finger CONSTANS-LIKE 5 | B-box zinc finger CONSTANS (Arabidopsis); Heading date1 (rice); GmCOL10 (soybean) | GI:351604446 | JP458797.1 | PK20924.1 | scaffold16587 | 1376 | 27...1337 |
| Zinc finger constans-like 5 | B-box zinc finger CONSTANS (Arabidopsis); Heading date1 (rice); GmCOL10 (soybean) | GI:351614347 | JP468648.1 | PK27784.1 | scaffold41868 | 257 | 0...257 |
| Zinc finger CONSTANS-LIKE 6 | B-box zinc finger CONSTANS (Arabidopsis); Heading date1 (rice); GmCOL10 (soybean) | GI:351629079 | JP481962.1 | PK18183.2 | scaffold12036 | 1784 | 1321...1784 |
|  |  |  |  |  | scaffold126972 | 1784 | 0...1456 |
|  |  |  |  |  | scaffold126972 | 1784 | 1231...1402 |
| Zinc finger CONSTANS-LIKE 6 | B-box zinc finger CONSTANS (Arabidopsis); Heading date1 (rice); GmCOL10 (soybean) | GI:351627832 | JP480715.1 | PK18183.3 | scaffold126972 | 1859 | 19...1858 |
| Zinc finger CONSTANS-LIKE 9-like | B-box zinc finger CONSTANS (Arabidopsis); Heading date1 (rice); GmCOL10 (soybean) | GI:351597768 | JP452308.1 | PK20565.1 | C32070077 | 1850 | 1078...1850 |
|  |  |  |  |  | C32075523 | 1850 | 140...862 |
|  |  |  |  |  | scaffold43158 | 1850 | 0...1091 |
|  |  |  |  |  | scaffold134962 | 1850 | 860...1091 |

**Supplementary Table 5.** Scaffolds with markers significantly associated to flowering time traits and sex determination containing fragments of flowering genes. PC1 and PC2 indicate the coordinates of each marker in the 3D scatter plot for the specific trait (**Figure 2** and **3**). See **Supplementary Table 4** for SeqName and Genbank accession codes of the flowering/sex related genes.

| Scaffold | Scaffold_marker | Trait | Location | Flowering / sex related genes | -log10P | Effect | PC1 | PC2 |
| --- | --- | --- | --- | --- | --- | --- | --- | --- |
| scaffold10005 | scaffold10005_124379 | sex_det | CRA | Flowering time control partial / Autonomous Flowering pathway; inhibiting FLC | 6.54 | 0.2556 | 0.03149 | -0.0018 |
|  |  | sex_det | FNPC | Flowering time control partial / Autonomous Flowering pathway; inhibiting FLC | 6.06 | 0.2818 | 0.03149 | -0.0018 |
|  |  | sex_det | VDS | Flowering time control partial / Autonomous Flowering pathway; inhibiting FLC | 5 | 0.2479 | 0.03149 | -0.0018 |
|  | scaffold10005_173186 | FL_FULL | VDS | Flowering time control partial / Autonomous Flowering pathway; inhibiting FLC | 9.99 | -0.3668 | 0.00372 | -0.0064 |
|  | scaffold10005_173201 | FL_FULL | VDS | Flowering time control partial / Autonomous Flowering pathway; inhibiting FLC | 9.61 | -0.3342 | 0.00709 | -0.0061 |
|  | scaffold10005_173315 | FL_FULL | VDS | Flowering time control partial / Autonomous Flowering pathway; inhibiting FLC | 6.83 | -0.2869 | 0.00275 | -0.0089 |
|  | scaffold10005_18123 | sex_det | VDS | Flowering time control partial / Autonomous Flowering pathway; inhibiting FLC | 4.28 | -0.2281 | -0.0187 | 0.00296 |
|  | scaffold10005_18192 | sex_det | VDS | Flowering time control partial / Autonomous Flowering pathway; inhibiting FLC | 4.16 | -0.2344 | -0.0156 | 0.01612 |
|  | scaffold10005_240020 | FL_FULL | VDS | Flowering time control partial / Autonomous Flowering pathway; inhibiting FLC | 4.05 | -0.2182 | 0.0119 | -0.0084 |
|  | scaffold10005_242128 | FL_FULL | VDS | Flowering time control partial / Autonomous Flowering pathway; inhibiting FLC | 4.24 | -0.1965 | -0.0005 | -0.0091 |
|  | scaffold10005_59528 | sex_det | VDS | Flowering time control partial / Autonomous Flowering pathway; inhibiting FLC | 4.74 | -0.1985 | -0.0125 | -0.0006 |
| scaffold10189 | scaffold10189_153123 | FL_FULL | FNPC | Zinc finger BED domain-containing RICESLEEPER 2-like | 5.52 | -0.2316 | 0.02211 | 0.00158 |
|  | scaffold10189_47734 | FL_FULL | FNPC | Zinc finger BED domain-containing RICESLEEPER 2-like | 4.13 | -0.1171 | 0.0167 | -0.0094 |
|  | scaffold10189_83355 | sex_det | FNPC | Zinc finger BED domain-containing RICESLEEPER 2-like | 4.21 | 0.5651 | 0.03006 | 0.00735 |
|  |  | sex_det | VDS | Zinc finger BED domain-containing RICESLEEPER 2-like | 4.05 | 0.5213 | 0.03006 | 0.00735 |
| scaffold102797 | scaffold102797_11002 | FL_FULL | FNPC | Floricaula leafy (flower and leaf development) | 5.26 | -0.1409 | 0.0166 | -0.005 |
|  | scaffold10879_25165 | FL_FULL | FNPC | Floricaula leafy (flower and leaf development) | 4.27 | -0.2614 | 0.01984 | 0.00582 |
|  | scaffold10879_25354 | FL_FULL | FNPC | Floricaula leafy (flower and leaf development) | 5.11 | -0.3006 | 0.02013 | 0.00382 |
| scaffold112970 | scaffold112970_6099 | VEG | CRA | MADS-box transcription factor | 4.07 | 0.1329 | 0.0146 | 0.03344 |
| scaffold11691 | scaffold11691_53534 | FL_BEGIN | VDS | Cryptochrome 1 family | 4.13 | -0.1723 | -0.0132 | 0.07664 |
|  |  | FL_FULL | VDS | Cryptochrome 1 family | 10.15 | -0.2761 | 0.01644 | -0.0066 |
|  |  | VEG | FNPC | Cryptochrome 1 family | 4.98 | -0.1543 | -0.0378 | 0.01694 |
|  | scaffold11691_54282 | FL_FULL | VDS | Cryptochrome 1 family | 5.16 | -0.2313 | 0.01613 | -0.0045 |
|  | scaffold11691_58162 | FL_FULL | VDS | Cryptochrome 1 family | 4.43 | -0.1317 | 0.01277 | 0.00866 |
| scaffold12036 | scaffold12036_40775 | FL_FULL | VDS | Zinc finger CONSTANS-LIKE 16 / Zinc finger CONSTANS-LIKE 6 | 7.08 | -0.3654 | 0.01678 | 0.00028 |
|  | scaffold12036_40806 | FL_FULL | VDS | Zinc finger CONSTANS-LIKE 16 / Zinc finger CONSTANS-LIKE 6 | 6.82 | -0.3553 | 0.01694 | 0.00069 |
|  | scaffold12036_77693 | FL_FULL | VDS | Zinc finger CONSTANS-LIKE 16 / Zinc finger CONSTANS-LIKE 6 | 4.63 | -0.284 | 0.00797 | -0.0047 |
| scaffold1342 | scaffold1342_129609 | FL_FULL | VDS | XAP5 CIRCADIAN TIMEKEEPER isoform X2 | 5.58 | -0.2042 | 0.0011 | -0.004 |
| scaffold13708 | scaffold13708_30759 | FL_FULL | FNPC | Zinc finger CONSTANS-LIKE 2 | 4.65 | -0.157 | 0.02018 | 0.00436 |
| scaffold16340 | scaffold16340_22562 | sex_det | CRA | bZIP transcription factor 16-like | 8.35 | 0.4564 | 0.03627 | 0.0015 |
|  |  | sex_det | FNPC | bZIP transcription factor 16-like | 13.56 | 0.6825 | 0.03627 | 0.0015 |
|  |  | sex_det | VDS | bZIP transcription factor 16-like | 4.4 | 0.4096 | 0.03627 | 0.0015 |
|  | scaffold16340_22609 | sex_det | FNPC | bZIP transcription factor 16-like | 5.67 | 0.5003 | 0.02702 | -0.0008 |
|  | scaffold16340_23032 | sex_det | CRA | bZIP transcription factor 16-like | 5.87 | 0.5161 | 0.03334 | -0.0196 |
|  |  | sex_det | FNPC | bZIP transcription factor 16-like | 5.66 | 0.5743 | 0.03334 | -0.0196 |
| scaffold16869 | scaffold16869_152682 | sex_det | CRA | Auxin response factor 2B-like isoform X1 | 5.27 | 0.228 | 0.02707 | -0.035 |
|  | scaffold16869_152716 | sex_det | CRA | Auxin response factor 2B-like isoform X1 | 5.41 | 0.2282 | 0.02683 | -0.0373 |
|  | scaffold16869_152825 | sex_det | CRA | Auxin response factor 2B-like isoform X1 | 4.17 | 0.1865 | 0.02607 | -0.0386 |
|  | scaffold16869_152913 | sex_det | CRA | Auxin response factor 2B-like isoform X1 | 5.91 | 0.2179 | 0.02671 | -0.0358 |
| scaffold1953 | scaffold1953_12600 | FL_FULL | VDS | SUPPRESSOR OF PHYA-105 1 (SPA1) | 4.59 | -0.2196 | 0.00533 | -0.0089 |
|  | scaffold1953_12605 | FL_FULL | VDS | SUPPRESSOR OF PHYA-105 1 (SPA1) | 4.57 | -0.2094 | 0.00571 | -0.0085 |
|  | scaffold1953_34320 | FL_FULL | FNPC | SUPPRESSOR OF PHYA-105 1 (SPA1) | 4.42 | -0.1355 | 0.01857 | -0.0063 |
| scaffold19844 | scaffold19844_7211 | VEG | CRA | Flowering LOCUS D (FLD) | 4.65 | 0.2743 | 0.01508 | 0.03277 |
|  | scaffold19844_7220 | VEG | CRA | Flowering LOCUS D (FLD) | 4.13 | 0.2623 | 0.01513 | 0.0322 |
|  | scaffold19844_7865 | VEG | CRA | Flowering LOCUS D (FLD) | 4.18 | 0.2594 | 0.01153 | 0.0323 |
| scaffold21255 | scaffold21255_72170 | sex_det | FNPC | Auxin response factor 5 | 4.57 | 0.4434 | 0.00913 | 0.02043 |
| scaffold21952 | scaffold21952_1421 | FL_BEGIN | VDS | Agamous-like MADS-box AGL93 | 4.51 | -0.1854 | -0.0119 | 0.00023 |
|  |  | VEG | VDS | Agamous-like MADS-box AGL93 | 4.43 | -0.1482 | -0.0031 | -0.0102 |
| scaffold2205 | scaffold2205_3731 | FL_FULL | VDS | B3 domain-containing transcription factor VRN1-like | 5.36 | -0.35 | 0.0177 | 0.0047 |
| scaffold24080 | scaffold24080_4722 | FL_FULL | FNPC | Flowering locus t-like4 | 8.2 | -0.2594 | 0.02254 | 0.0031 |
|  | scaffold24080_5339 | FL_FULL | FNPC | Flowering locus t-like4 | 7.85 | -0.2524 | 0.02231 | 0.00224 |
| scaffold2448 | scaffold2448_65390 | FL_BEGIN | CRA | bZIP transcription factor 27-like | 4.22 | 0.3095 | 0.04511 | 0.01279 |
|  |  | FL_FULL | FNPC | bZIP transcription factor 27-like | 4.46 | 0.1746 | -0.0015 | 0.05282 |
|  |  | sex_det | CRA | bZIP transcription factor 27-like | 4.73 | 0.4219 | 0.01089 | 0.07423 |
|  |  | VEG | CRA | bZIP transcription factor 27-like | 5.68 | 0.3202 | 0.01962 | 0.04048 |
| scaffold29022 | scaffold29022_6971 | VEG | CRA | Zinc finger BED domain-containing RICESLEEPER 2-like | 4.25 | 0.1353 | 0.01591 | 0.02179 |
| scaffold31176 | scaffold31176_27420 | sex_det | CRA | DELLA RGL1-like / Repressor of the gibberellin (GA) signaling pathway. Regulates the floral development. | 5.21 | 0.4508 | 0.02963 | -0.0158 |
|  | scaffold31176_27484 | sex_det | CRA | DELLA RGL1-like / Repressor of the gibberellin (GA) signaling pathway. Regulates the floral development. | 5.12 | 0.4423 | 0.02948 | -0.017 |
|  | scaffold31176_27674 | sex_det | CRA | DELLA RGL1-like / Repressor of the gibberellin (GA) signaling pathway. Regulates the floral development. | 5.57 | 0.3818 | 0.02764 | -0.0192 |
| scaffold3201 | scaffold3201_17293 | FL_BEGIN | FNPC | Flowering locus t-like3 | 4.45 | -0.1117 | -0.019 | 0.06911 |
|  |  | FL_FULL | VDS | Flowering locus t-like3 | 5.69 | -0.1538 | 0.0151 | -0.013 |
|  |  | VEG | FNPC | Flowering locus t-like3 | 5.33 | -0.1034 | -0.0369 | 0.00956 |
|  | scaffold3201_18032 | FL_FULL | VDS | Flowering locus t-like3 | 6.4 | -0.1549 | 0.0152 | -0.0116 |
|  | scaffold3201_6178 | FL_BEGIN | FNPC | Flowering locus t-like3 | 5.37 | -0.1943 | -0.0193 | 0.07752 |
|  |  | FL_BEGIN | VDS | Flowering locus t-like3 | 5.21 | -0.202 | -0.0193 | 0.07752 |
|  |  | FL_FULL | FNPC | Flowering locus t-like3 | 6.16 | -0.1631 | 0.01684 | -0.0132 |
|  |  | FL_FULL | VDS | Flowering locus t-like3 | 12.22 | -0.3074 | 0.01684 | -0.0132 |
|  |  | VEG | FNPC | Flowering locus t-like3 | 6.67 | -0.1837 | -0.0405 | 0.01219 |
|  |  | VEG | VDS | Flowering locus t-like3 | 5.34 | -0.165 | -0.0405 | 0.01219 |
|  | scaffold3201_6224 | sex_det | CRA | Flowering locus t-like3 | 4.29 | -0.1902 | -0.0126 | 0.03936 |
|  |  | FL_BEGIN | FNPC | Flowering locus t-like3 | 4.9 | -0.1831 | -0.018 | 0.07718 |
|  |  | FL_BEGIN | VDS | Flowering locus t-like3 | 4.45 | -0.1863 | -0.018 | 0.07718 |
|  |  | FL_FULL | FNPC | Flowering locus t-like3 | 4.55 | -0.1448 | 0.01666 | -0.0116 |
|  |  | FL_FULL | VDS | Flowering locus t-like3 | 9.86 | -0.2839 | 0.01666 | -0.0116 |
|  |  | VEG | FNPC | Flowering locus t-like3 | 6.03 | -0.1727 | -0.0397 | 0.01307 |
|  |  | VEG | VDS | Flowering locus t-like3 | 4.57 | -0.1524 | -0.0397 | 0.01307 |
| scaffold33564 | scaffold33564_4933 | FL_FULL | VDS | Phytochrome E | 6.19 | -0.4735 | 0.01674 | -0.0014 |
| scaffold35877 | scaffold35877_37494 | sex_det | CRA | Probable lysine-specific demethylase ELF6 /Circadian clock, photoperiodism, Flowering | 4.26 | 0.1987 | 0.02812 | -0.0356 |
| scaffold42686 | scaffold42686_5560 | FL_FULL | FNPC | Phytochrome A | 9.56 | -0.2641 | 0.02222 | 0.00379 |
| scaffold4343 | scaffold4343_57422 | VEG | CRA | Squamosa promoter-binding 1-like (SPL1-like) | 4.48 | 0.1384 | 0.01956 | 0.01915 |
|  | scaffold4343_57467 | FL_BEGIN | CRA | Squamosa promoter-binding 1-like (SPL1-like) | 4.12 | 0.1574 | 0.03066 | -0.0185 |
|  |  | VEG | CRA | Squamosa promoter-binding 1-like (SPL1-like) | 4.53 | 0.1468 | 0.02111 | 0.01953 |
|  | scaffold4343_57592 | FL_BEGIN | CRA | Squamosa promoter-binding 1-like (SPL1-like) | 5.68 | 0.1983 | 0.03314 | -0.0122 |
|  |  | FL_FULL | CRA | Squamosa promoter-binding 1-like (SPL1-like) | 5.39 | 0.1797 | -0.0037 | 0.03932 |
|  |  | FL_FULL | FNPC | Squamosa promoter-binding 1-like (SPL1-like) | 4.07 | 0.0911 | -0.0037 | 0.03932 |
|  |  | VEG | CRA | Squamosa promoter-binding 1-like (SPL1-like) | 6.23 | 0.1843 | 0.02013 | 0.02382 |
|  | scaffold4343_57626 | FL_BEGIN | CRA | Squamosa promoter-binding 1-like (SPL1-like) | 4.09 | 0.1572 | 0.03075 | -0.0189 |
|  |  | VEG | CRA | Squamosa promoter-binding 1-like (SPL1-like) | 4.5 | 0.1465 | 0.02121 | 0.01941 |
|  | scaffold4343_57637 | FL_BEGIN | CRA | Squamosa promoter-binding 1-like (SPL1-like) | 4.33 | 0.162 | 0.03098 | -0.0189 |
|  |  | VEG | CRA | Squamosa promoter-binding 1-like (SPL1-like) | 4.74 | 0.1507 | 0.02135 | 0.01961 |
|  | scaffold4343_57682 | FL_BEGIN | CRA | Squamosa promoter-binding 1-like (SPL1-like) | 4.31 | 0.1639 | 0.03113 | -0.0191 |
|  |  | FL_FULL | CRA | Squamosa promoter-binding 1-like (SPL1-like) | 4.1 | 0.1486 | -0.0047 | 0.03476 |
|  |  | VEG | CRA | Squamosa promoter-binding 1-like (SPL1-like) | 4.74 | 0.1529 | 0.02143 | 0.01964 |
|  | scaffold4343_58092 | FL_BEGIN | CRA | Squamosa promoter-binding 1-like (SPL1-like) | 4.53 | 0.1566 | 0.03317 | -0.0184 |
|  |  | FL_FULL | CRA | Squamosa promoter-binding 1-like (SPL1-like) | 4.86 | 0.1513 | -0.0044 | 0.03771 |
|  |  | VEG | CRA | Squamosa promoter-binding 1-like (SPL1-like) | 4.82 | 0.1434 | 0.02172 | 0.02123 |
| scaffold4564 | scaffold4564_139237 | FL_FULL | VDS | Flowering locus t-like1 | 4.93 | -0.1302 | 0.01377 | -0.014 |
|  | scaffold4564_139387 | FL_FULL | VDS | Flowering locus t-like1 | 4.86 | -0.1369 | 0.01398 | -0.0129 |
|  | scaffold4564_167771 | FL_FULL | VDS | Flowering locus t-like1 | 4.16 | -0.1289 | 0.01035 | -0.012 |
|  | scaffold4564_170596 | FL_FULL | VDS | Flowering locus t-like1 | 7.08 | -0.2895 | 0.0048 | -0.0026 |
|  | scaffold4564_213551 | FL_FULL | VDS | Flowering locus t-like1 | 4.48 | -0.1404 | 0.01121 | -0.0028 |
|  | scaffold4564_214104 | FL_FULL | VDS | Flowering locus t-like1 | 4.19 | -0.1811 | 0.01221 | -0.0023 |
|  | scaffold4564_222585 | FL_FULL | VDS | Flowering locus t-like1 | 4.09 | -0.1078 | 0.01062 | -0.0012 |
|  | scaffold4564_222610 | FL_FULL | VDS | Flowering locus t-like1 | 4.12 | -0.1141 | 0.01022 | -0.0015 |
|  | scaffold4564_237296 | FL_FULL | VDS | Flowering locus t-like1 | 4.09 | -0.1588 | 0.0126 | 0.00161 |
|  | scaffold4564_237599 | FL_BEGIN | VDS | Flowering locus t-like1 | 6.79 | -0.1759 | -0.0126 | 0.07365 |
|  |  | FL_FULL | VDS | Flowering locus t-like1 | 9.23 | -0.2472 | 0.0139 | -0.0064 |
|  |  | VEG | VDS | Flowering locus t-like1 | 6.7 | -0.141 | -0.0332 | 0.01346 |
|  | scaffold4564_241072 | FL_FULL | VDS | Flowering locus t-like1 | 4.4 | -0.1461 | 0.01166 | 0.00394 |
|  | scaffold4564_241081 | FL_FULL | VDS | Flowering locus t-like1 | 4.61 | -0.1439 | 0.01232 | 0.00349 |
|  | scaffold4564_58881 | FL_FULL | VDS | Flowering locus t-like1 | 7.21 | -0.1642 | 0.01588 | -0.0104 |
|  |  | VEG | FNPC | Flowering locus t-like1 | 4.41 | -0.0969 | -0.0379 | 0.01278 |
|  | scaffold4564_59925 | FL_BEGIN | FNPC | Flowering locus t-like1 | 4.17 | -0.1388 | -0.0191 | 0.0732 |
|  |  | FL_FULL | FNPC | Flowering locus t-like1 | 5.84 | -0.1299 | 0.0163 | -0.0121 |
|  |  | FL_FULL | VDS | Flowering locus t-like1 | 7.06 | -0.196 | 0.0163 | -0.0121 |
|  |  | VEG | FNPC | Flowering locus t-like1 | 5.39 | -0.1347 | -0.0392 | 0.01126 |
|  | scaffold4564_59963 | FL_FULL | FNPC | Flowering locus t-like1 | 5.53 | -0.1332 | 0.01656 | -0.0112 |
|  |  | FL_FULL | VDS | Flowering locus t-like1 | 5.49 | -0.1881 | 0.01656 | -0.0112 |
|  |  | VEG | FNPC | Flowering locus t-like1 | 5.09 | -0.1377 | -0.0395 | 0.01188 |
|  | scaffold4564_60333 | FL_FULL | FNPC | Flowering locus t-like1 | 4.3 | -0.1157 | 0.01588 | -0.01 |
|  |  | VEG | FNPC | Flowering locus t-like1 | 4.84 | -0.1225 | -0.0377 | 0.01155 |
|  | scaffold4564_87593 | FL_FULL | FNPC | Flowering locus t-like1 | 4.5 | -0.1274 | 0.01716 | -0.0051 |
|  |  | FL_FULL | VDS | Flowering locus t-like1 | 7.45 | -0.2116 | 0.01716 | -0.0051 |
| scaffold46774 | scaffold46774_18772 | FL_BEGIN | CRA | Ultraviolet-B receptor UVR8 | 4.23 | 0.337 | 0.04765 | 0.01883 |
|  |  | FL_FULL | CRA | Ultraviolet-B receptor UVR8 | 4.07 | 0.3074 | 0.00012 | 0.05528 |
|  |  | VEG | CRA | Ultraviolet-B receptor UVR8 | 4.57 | 0.3111 | 0.01722 | 0.04274 |
| scaffold5190 | scaffold5190_181239 | FL_FULL | FNPC | Gibberellin-20 oxidase / gibberellin 2-beta-dioxygenase 1 / agamous-like MADS-box AGL6 | 5.35 | -0.2367 | 0.02115 | 0.00092 |
|  | scaffold5190_181422 | FL_FULL | VDS | Gibberellin-20 oxidase / gibberellin 2-beta-dioxygenase 1 / agamous-like MADS-box AGL6 | 4.39 | -0.177 | 0.01184 | 0.00274 |
|  | scaffold5190_181519 | FL_BEGIN | VDS | Gibberellin-20 oxidase / gibberellin 2-beta-dioxygenase 1 / agamous-like MADS-box AGL6 | 4.12 | -0.3265 | -0.0065 | 0.0914 |
|  |  | FL_FULL | FNPC | Gibberellin-20 oxidase / gibberellin 2-beta-dioxygenase 1 / agamous-like MADS-box AGL6 | 4.7 | -0.2608 | 0.02148 | 0.00697 |
|  |  | FL_FULL | VDS | Gibberellin-20 oxidase / gibberellin 2-beta-dioxygenase 1 / agamous-like MADS-box AGL6 | 8.52 | -0.5238 | 0.02148 | 0.00697 |
|  | scaffold5190_189172 | VEG | FNPC | Probable UDP-N-acetylglucosamine--peptide N-acetylglucosaminyltransferase SPINDLY / GA balance | 4.32 | -0.0909 | -0.0274 | 0.01139 |
|  | scaffold5190_189180 | FL_BEGIN | FNPC | Gibberellin-20 oxidase / gibberellin 2-beta-dioxygenase 1 / agamous-like MADS-box AGL6 | 4.09 | -0.1016 | -0.0103 | 0.06071 |
|  |  | VEG | FNPC | Probable UDP-N-acetylglucosamine--peptide N-acetylglucosaminyltransferase SPINDLY / GA balance | 4.9 | -0.0949 | -0.0277 | 0.01137 |
|  | scaffold5190_25929 | FL_FULL | FNPC | Gibberellin-20 oxidase / gibberellin 2-beta-dioxygenase 1 / agamous-like MADS-box AGL6 | 4.3 | -0.2367 | 0.01962 | 0.00254 |
|  | scaffold5190_43475 | sex_det | FNPC | Probable UDP-N-acetylglucosamine--peptide N-acetylglucosaminyltransferase SPINDLY / GA balance | 4.9 | 0.2603 | 0.02928 | -0.0262 |
| scaffold64764 | scaffold64764_26108 | FL_FULL | VDS | Zinc finger BED domain-containing RICESLEEPER 2-like | 6.17 | -0.5012 | 0.0122 | -0.0017 |
| scaffold75641 | scaffold75641_7087 | FL_FULL | VDS | leucine-rich repeat receptor-like serine threonine- kinase BAM3 isoform X1 | 6.07 | -0.3262 | 0.01968 | 0.0038 |
| scaffold9737 | scaffold9737_2615 | FL_FULL | FNPC | Probable UDP-N-acetylglucosamine--peptide N-acetylglucosaminyltransferase SPINDLY / GA balance | 6 | -0.2497 | 0.02201 | 0.00113 |
|  |  | FL_FULL | VDS | Probable UDP-N-acetylglucosamine--peptide N-acetylglucosaminyltransferase SPINDLY / GA balance | 4.79 | -0.3508 | 0.02201 | 0.00113 |
|  | scaffold9737_2638 | FL_FULL | FNPC | Probable UDP-N-acetylglucosamine--peptide N-acetylglucosaminyltransferase SPINDLY / GA balance | 4.65 | -0.2047 | 0.02068 | 0.00008 |
|  |  | FL_FULL | VDS | Probable UDP-N-acetylglucosamine--peptide N-acetylglucosaminyltransferase SPINDLY / GA balance | 4.09 | -0.2978 | 0.02068 | 0.00008 |

**Reference Supplementary tables**

Petit, J., Salentijn, E.M.J., Paulo, M.J., Thourminot, C., Van Dinter, B.J., Magagnini, G., Gusovius, H.J., Tang, K., Amaducci, S., Wang, S., Uhrlaub, B., Mussig, J., and Trindade, L.M. (2020). Genetic variability of morphological, flowering and biomass quality traits in hemp (*Cannabis sativa* L.). *Front. Plant Sci.* 10. doi: 10.3389/fpls.2020.00102.

van Bakel, H., Stout, J.M., Cote, A.G., Tallon, C.M., Sharpe, A.G., Hughes, T.R., and Page, J.E. (2011). The draft genome and transcriptome of *Cannabis sativa*. *Genome Biol.* 12, R102. doi: 10.1186/gb-2011-12-10-r102.
